# Host associations and genetic diversity of bat flies (Diptera: Nycteribiidae and Streblidae) in bats from Thailand

**DOI:** 10.1186/s13071-025-06814-y

**Published:** 2025-05-24

**Authors:** Dimas Novianto, Siwaporn Tuangpermsub, Thongchai Ngamprasertwong, Morakot Kaewthamasorn

**Affiliations:** 1 Center of Excellence in Veterinary Parasitology, Department of Pathology, Faculty of Veterinary Science, Chulalongkorn University, Bangkok, 10330 Thailand; 2 Veterinary Pathobiology Graduate Program, Department of Pathology, Faculty of Veterinary Science, Chulalongkorn University, Bangkok, 10330 Thailand; 3 Department of Biology, Faculty of Science, Chulalongkorn University, Bangkok, 10330 Thailand

**Keywords:** Bat flies, Host-vector interaction, Phylogenetics, Species delimitation, Thailand

## Abstract

**Background:**

Bat flies belong to the order Diptera and superfamily Hippoboscoidea. They can be divided into two families, Streblidae and Nycteribiidae, which collectively encompass 239 and 280 species worldwide, respectively. In Thailand, 43 species of Nycteribiidae and 16 species of Streblidae have been documented. Despite their diversity, the molecular characteristics and host-parasite interactions of these ectoparasites remain poorly understood.

**Methods:**

During a bat survey conducted between 2019 and 2022, bat flies were collected across eight sites in three provinces of Thailand. Morphological identification was performed using identification keys and a bat fly checklist endemic to Thailand. DNA barcoding targeted to the mitochondrial Cox1 and nuclear 28S rRNA genes was utilized. Infestation patterns were analyzed in relation to host sex, sampling site, and physiological status. Species identification was confirmed via BLASTN searches, and species delimitation was conducted using the ASAP algorithm under three substitution models. Phylogenetic relationships were inferred using Maximum Likelihood methods, while genetic variation was assessed through TCS haplotype network analysis. Tripartite network analysis was employed to examine site-host-parasite associations.

**Results:**

A total of 1,042 bats, representing 28 species, were captured during the study, of which 298 individuals (28.59%) were infested with bat flies. In total, 773 bat flies were collected, comprising 737 from the family Streblidae and 36 from Nycteribiidae. Morphological and molecular analyses identified three genera—*Raymondia*, *Brachytarsina*, and *Nycteribia*—along with seven hypothetical species. Phylogenetic reconstruction using mitochondrial (*Cox*1) and nuclear (28S rRNA) gene markers revealed distinct clades within each genus, underscoring substantial genetic diversity. Haplotype analyses identified 18 haplotypes in *Raymondia*, six in *Brachytarsina*, and two in *Nycteribia*, with evidence of site-specific host-parasite associations. Infestation rates varied by host species, sex, and location, with larger bat populations demonstrating higher infestation intensities. *Raymondia* sp. 1 is the most frequently encountred species an predominantly infested *Hipposideros gentilis*.

**Conclusions:**

This study provides the first molecular characterization of bat fly diversity in Thailand, revealing their genetic complexity, taxonomy, host specificity, and ecological interactions. The findings establish a crucial foundation for further research concerning the biodiversity, host-parasite dynamics, and zoonotic risks associated with bat flies.

**Graphical Abstract:**

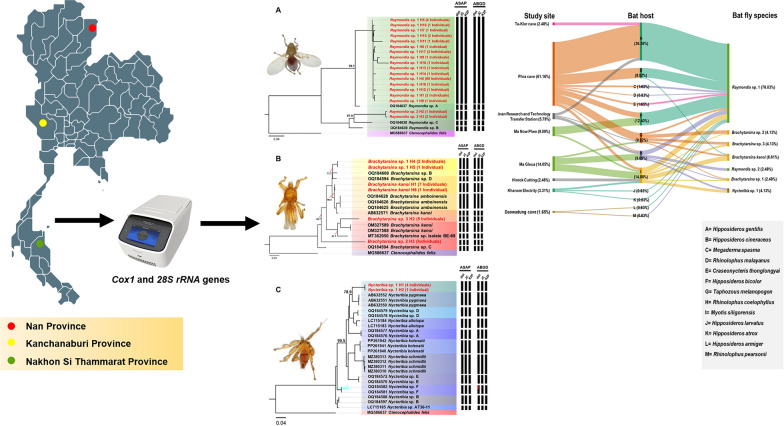

**Supplementary Information:**

The online version contains supplementary material available at 10.1186/s13071-025-06814-y.

## Background

Bats (order Chiroptera) represent one of the most diverse and ecologically significant mammalian groups, comprising over 1482 species globally [1]. They fulfill vital ecosystem functions, including pollination, seed dispersal, pest control, and nutrient cycling, thereby supporting forest regeneration, agricultural productivity, and the stability of cave and forest ecosystems [2]. Beyond their ecological importance, bats serve as reservoirs for various pathogens, including those with zoonotic potential, maintaining natural transmission cycles to other mammals [3]. However, studying bats poses significant challenges due to their nocturnal behavior and protected status, which restrict capture and researcher access. In addition to harboring viruses, bacteria, and internal parasites, bats support a diverse range of ectoparasites, such as ticks (families Argasidae and Ixodidae) and bat flies (Diptera: Nycteribiidae and Streblidae) [4, 5]. These ectoparasites can affect bat health, behavior, and disease susceptibility and increase the risk of pathogen spillover to other species [3]. Furthermore, recent studies have demonstrated that bats are capable of long-distance migrations from their roosting sites [6]. This raises the hypothesis that migrating bats may facilitate the geographic spread of ectoparasites by transporting them to new roosting sites, thereby contributing to the dissemination of these vectors and the organisms they harbor across regions.

Bat flies are obligate hematophagous ectoparasites belonging to the superfamily Hippoboscoidea, which feed exclusively on bat blood. They reproduce via viviparous puparity, a reproductive strategy that ensures the development of advanced larvae that are ready for pupation [7, 8]. The two families of bat flies, Streblidae and Nycteribiidae, exhibit distinct morphological characteristics; Streblidae possess functional wings, whereas Nycteribiidae are wingless with arachnid-like bodies [7]. Their adaptations, including sclerotized integuments, protective setae, and backward-pointing claws, facilitate secure attachment to hosts and enable movement within fur and wing membranes [9]. Globally, 239 species of Streblidae and 280 species of Nycteribiidae have been documented, with Thailand currently hosting 59 recognized species (43 Nycteribiidae and 16 Streblidae) that parasitize 63 bat species [5, 10]. Despite their remarkable diversity, the taxonomy of bat flies remains challenging because of their morphological similarities, the limited availability of reliable taxonomic keys, and the scarcity of comprehensive genomic studies.

Information on bat flies, their infestation patterns, and their relationships with bat hosts has been extensively documented in regions such as Mexico, Brazil, Nigeria, Kenya, Hong Kong, and Singapore [11–18]. However, these topics remain largely unexplored in Thailand because of the limited scope of bat studies conducted within the country. Investigating bat fly communities, including their diversity, abundance, and host specificity, is critical for advancing our understanding of host-parasite-pathogen interactions. Host-parasite relationships are shaped by various factors, including roosting habits, body size, social behavior, roost microclimate, vegetation type, and seasonal variations [19–22]. Notably, some bat roosting sites, such as attics within human-made structures, bring bats into closer proximity to humans than is often realized. Furthermore, the vectorial roles of bat flies are conspicuously understudied compared to other medically important arthropods, such as mosquitoes and ticks [23, 24].

Identification of bat fly species is essential for understanding their diversity, ecological functions, and evolutionary relationships. Morphological identification presents challenges owing to phenotypic similarities among species and the presence of cryptic species, which complicates taxonomic resolution. Molecular techniques, such as DNA barcoding using the mitochondrial cytochrome oxidase I (*Cox1*) gene, have demonstrated efficacy in enhancing species identification and phylogenetic analyses [25]. However, the *Cox1* database for bat flies remains incomplete, necessitating the expansion of genetic datasets to improve taxonomic studies. Verrett et al. [16] elucidated the value of integrating nuclear markers, such as the 28S rRNA gene, with *Cox1* for phylogenetic studies in bat flies. By combining 28S rRNA with *Cox1*, researchers can construct robust phylogenies that benefit from both nuclear and mitochondrial data, thereby mitigating the risk of misleading conclusions associated with relying on a single genetic marker.

The objective of this study was to investigate the diversity, distribution, and host associations of bat flies in bats in Thailand, focusing on their infestation patterns, host specificity, and ecological relationships. To address taxonomic challenges, this research employed a combination of morphological identification and molecular techniques, including mitochondrial cytochrome oxidase I (*Cox1*) and nuclear 28S rRNA markers, to enhance species identification and phylogenetic analysis.

## Methods

### Sampling site and sample collection

**Fig. 1:**
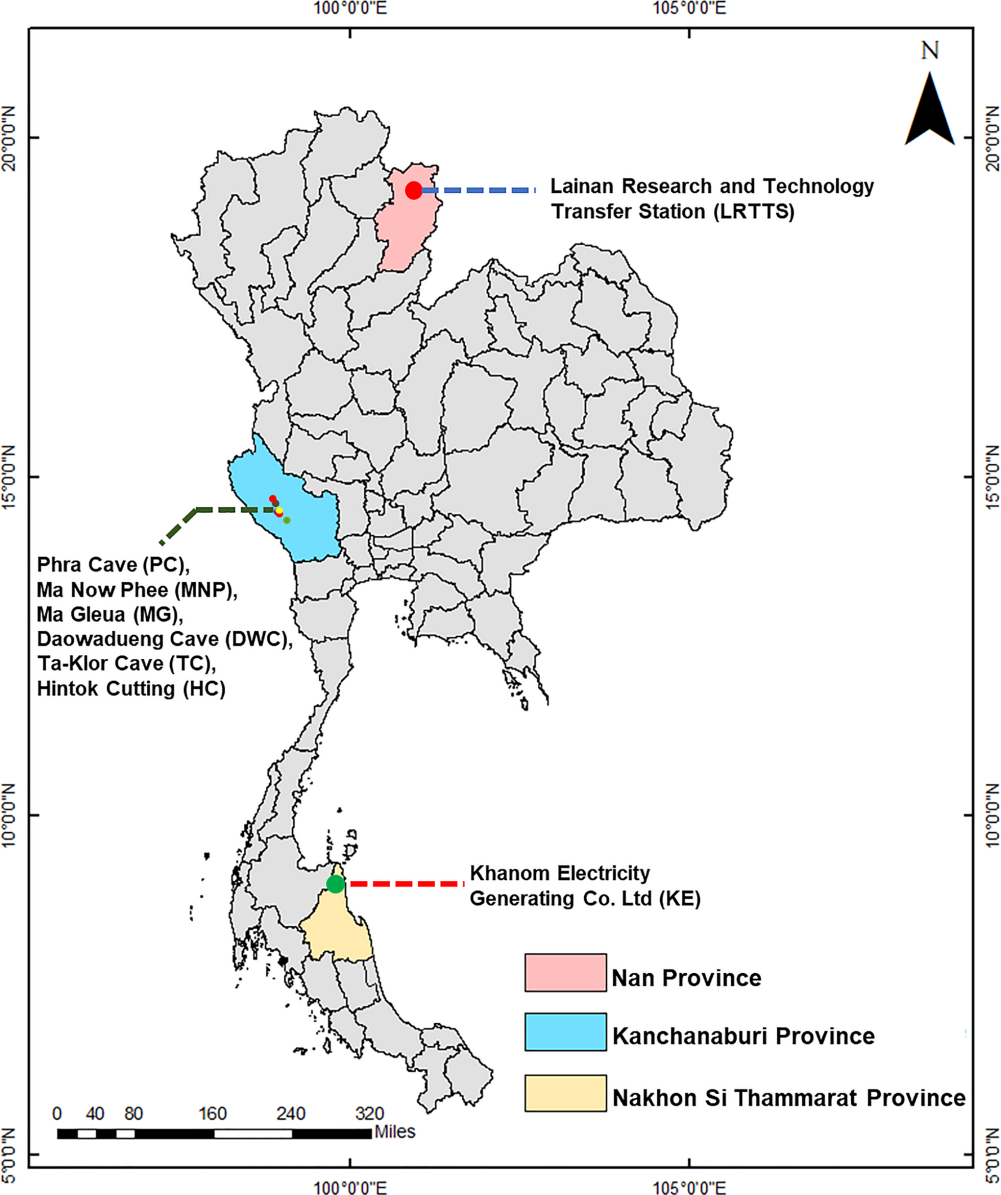
Geographical map depicting the locations of eight sampling sites across three provinces in Thailand. The map was generated using ArcGIS version 10.8

This study was conducted as part of a bat parasite survey previously described by Arnuphapprasert et al. [26] and Riana et al. [27]. Bats and their associated bat flies were collected from eight locations across three provinces in Thailand. The sampling sites included the Lainan Research and Technology Transfer Station (LRTTS; GPS coordinates: 18°33′14.5"N, 100°47′32.8"E) in Nan Province, Phra Cave (PC; 14°24′36.6"N, 98°51′13.5"E), Ma Now Phee Cave (MNP; 14°21′19.6"N, 98°56′14.1"E), Ma Gleua Cave (MG; 14°21′31.9"N, 98°56′22.8"E), Daowadung Cave (DWC; 14°27′59"N, 98°49′51"E), Ta-Klor Cave (TC; 14°20′34"N, 98°57′28"E), and Hintok Cutting (HC; 14°21′52"N, 98°56′17"E) in Kanchanaburi Province and the Khanom Electricity Generating Co., Ltd. (KE; 9°13′48.3″N, 99°51′15.6"E) in Nakhon Si Thammarat Province (Fig. 1). Bat reproductive status was recorded using the following abbreviations: AD = adult, AN = adult nulliparous, JU = juvenile, LA = lactating, NR = nonreproductive, NU = nulliparous, PA = parous, and PR = pregnant. Bat flies were collected during surveys conducted between 2019 and 2022. Using fine forceps, each bat fly was carefully removed from its host, individually preserved in a vial containing 96% ethanol, and stored at − 20 °C until further analysis. The host species and sex were recorded for each sample to facilitate further investigations.

### Morphological identification, DNA extraction, and amplification of Cox1 and 28S rRNA genes of bat flies

The collected bat flies were taxonomically classified to the genus level using the checklist of Nycteribiid and Streblid bat flies endemic to Thailand [5]. Specimens were photographed using a stereomicroscope (Olympus SZ61 series, Japan).

Genomic DNA (gDNA) was extracted from the entire bodies of individual bat flies using a NucleoSpin® Tissue kit (Macherey-Nagel, Germany) following the manufacturer’s protocol with a modification in the elution step. To ensure high gDNA concentration, the volume of pre-heated BE buffer (70 °C) was reduced from 100 µl to 60 µl before elution through the column. The extracted gDNA was stored at − 20 °C until further use. Polymerase chain reaction (PCR) was employed to amplify the mitochondrial cytochrome oxidase I (*Cox1*) gene and the nuclear 28S rRNA gene. The primers and PCR protocols used for amplification are detailed in Supplementary Table S1. Amplified PCR products were separated on a 1.5% (w/v) agarose gel stained with RedSafe™ (Intron Biotechnology, Korea) and visualized under a UV transilluminator.

### DNA Sequencing

PCR products free of nonspecific bands were treated with ExoSAP-IT™ (Applied Biosystems, Lithuania) to eliminate residual primers and nucleotides. For products exhibiting nonspecific bands, target bands were isolated through agarose gel electrophoresis, excised, and purified using the NucleoSpin® Gel and PCR Clean-up Kit (Macherey-Nagel, Germany) following the manufacturer’s protocol. The purified products were then sequenced bidirectionally using the Sanger sequencing method, with sequencing services provided by U2Bio Co., Ltd. (https://www.u2bio.co.th/home).

### Sequence analysis of COI and 28S rRNA genes of bat flies

Sequencing results with high-quality chromatograms were rigorously evaluated through a series of quality control measures, including visual inspection, manual editing, trimming, and the assembly of consensus sequences from forward and reverse reads using BioEdit software [28]. Chromatograms of low quality or with ambiguities were excluded from further analyses to ensure data reliability. For protein-coding genes, sequences were translated into amino acid sequences to verify the absence of internal stop codons and maintain the correct reading frames. Subsequently, alignments were conducted using the MUSCLE algorithm in MEGA11 [29], ensuring optimal sequence alignment accuracy. The finalized consensus sequences were cross-referenced for nucleotide identity against the GenBank database (https://www.ncbi.nlm.nih.gov/) using the Basic Local Alignment Search Tool (BLASTN), providing robust validation of sequence annotations.

### Phylogenetic analysis and species delimitation

For phylogenetic analysis, *Cox1* and 28S rRNA gene sequences of bat flies obtained from GenBank were aligned and standardized to uniform lengths using BioEdit v7.2.5. Maximum likelihood (ML) phylogenetic trees were constructed in IQ-TREE v2, with the best-fitting substitution models identified via ModelFinder [30]. The selected models were GTR + F + I + G4 for *Cox1* and F81 + F + G4 for 28S rRNA. Node support values were calculated using ultrafast bootstrapping with 1000 replications. The resulting phylogenetic trees were visualized in FigTree v1.4.4. Species delimitation based on *Cox1* sequences was performed using two complementary methods: Assemble Species by Automatic Partitioning (ASAP) and Automatic Barcode Gap Discovery (ABGD). ASAP analyses were conducted via the web server (https://bioinfo.mnhn.fr/abi/public/asap/asapweb.html) using three substitution models: p-distance, JC69, and K2P. Each analysis was performed in 10 replicates, with the species partition exhibiting the lowest ASAP score selected to ensure consistent results. ABGD analyses were conducted on the web server (https://bioinfo.mnhn.fr/abi/public/abgd/) using default parameters, including a maximum intraspecific distance (Pmax) of 0.1 and a minimum (Pmin) of 0.001. Both approaches yielded complementary insights into species delimitation, enhancing the reliability of the findings [31, 32].

### Genetic diversity analysis

The genetic diversity of *Cox1* and 28S rRNA gene sequences from bat flies was assessed using DnaSP v5.10.01 (http://www.ub.edu/dnasp/). Key parameters analyzed included the number of polymorphic sites, haplotype count, haplotype diversity, nucleotide diversity, and neutrality tests such as Tajima’s D and Fu and Li’s D statistics [33]. To investigate genetic relationships further, haplotype networks were generated using the TCS algorithm in PopART v1.7 [34], incorporating nucleotide sequences obtained in this study alongside previously published datasets (Table S2).

### Data analysis

Infestation patterns of bat flies were analyzed across bat species, sex, and physiological statuses using descriptive statistics. To evaluate the influence of these factors on the extent of bat fly infestations, a Poisson generalized linear model (GLM) was applied, with model selection based on Akaike information criterion (AIC) and Bayesian information criterion (BIC). The independent variables (predictors) included bat species, sex, physiological status, and study site, while the dependent variable (response) was the number of bat flies per bat. Additionally, Spearman correlation analysis was performed to examine the relationship between bat population size and infestation intensity. A tripartite network analysis was conducted using 121 individual bat fly sequences, incorporating data on geographic locations, bat host species, and bat fly species to delineate interaction subsets and uncover ecological patterns.

## Results

### Streblid and nyteribiid fly infestation in bat hosts

**Fig. 2:**
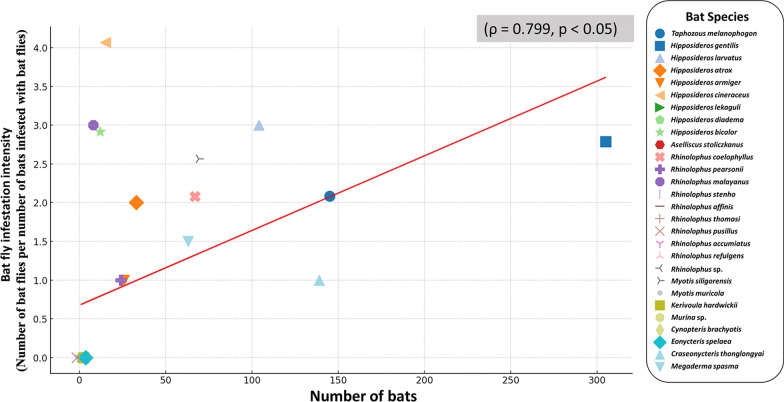
Relationship between bat population size and the number of infested bats. Each point represents a bat species, with the x-axis indicating the total number of bats and the y-axis representing the corresponding number of infested individuals

Between 2019 and 2022, a total of 1042 bats representing 28 species across seven families were examined for bat fly infestations (Table 1). The results revealed that 298 bats (28.59%) were infested with bat flies. Streblidae were the predominant ectoparasite (n = 737), with *Hipposideros bicolor* and *H. cineraceus* exhibiting the highest infestation rates at 100% (12 out of 12 *H. bicolor* and 15 out of 15 *H. cineraceus* bats, respectively). *Rhinolophus coelophyllus* showed mixed infestations (streblid and nycteribiid), with an overall infestation rate of 56.7% (36/67 bats), predominantly by streblid-only (50.7%, 34/67), while nycteribiid-only and mixed infestations each accounted for 2.9% (2/67). *Hipposideros gentilis* exhibited a Streblidae-specific infestation rate of 47.5% (145/305), while *Myotis siligorensis* displayed a more diverse infestation profile, with 13% (9/69) attributed to streblid-only, 10.1% (7/69) to nycteribiid-only, and 20.3% to mixed infestations (14/69). Among species with low infestation rates, *Craseonycteris thonglongyai*, the smallest bat species in this study, had an infestation rate of 1.44%, with only two out of 139 individuals infested by bat flies. Similarly, *Megaderma spasma* exhibited a low infestation rate of 1.58%, with two out of 63 individuals infested. These two species demonstrated the lowest infestation rates among those studied, indicating limited exposure to bat flies. The details of bat fly infestations observed in this study are provided in Table S3. Additionally, this study identified a strong positive correlation (ρ = 0.79, *p* < 0.05) between bat population size and infestation intensity of bat flies (Fig. 2).

**Table 1:** Bat species composition, abundance, and infestation rates across eight study sites in Thailand (2019–2022)

Family	Genus	*Species*	Number of bats	Number of bats infested by bat fly			Total proportion infestation (%)	Number of bat fly	
				Streblidae	Nycteribiidae	Mixed infestation*		Streblidae	Nycteribiidae
Emballonuridae	*Taphozous*	*Taphozous melanopogon*	145	47	0	0	32.41	98	0
Hipposideridae	*Hipposideros*	*Hipposideros gentilis*	305	145	0	0	47.54	404	0
		*Hipposideros larvatus*	104	1	0	0	0.96	3	0
		*Hipposideros atrox*	33	3	0	0	9.09	6	0
		*Hipposideros armiger*	26	1	0	0	3.85	1	0
		*Hipposideros cineraceus*	15	15	0	0	100	61	0
		*Hipposideros lekaguli*	5	0	0	0	0	0	0
		*Hipposideros diadema*	2	0	0	0	0	0	0
		*Hipposideros bicolor*	12	12	0	0	100	35	0
	*Aselliscus*	*Asellicus stoliczlcanus*	2	0	0	0	0	0	0
Rhinolophidae	*Rhinolophus*	*Rhinolophus coelophyllus*	67	34	2	2	56.72	74	5
		*Rhinolophus pearsonii*	24	1	0	0	4.17	1	0
		*Rhinolopus malayanus*	8	1	0	0	12.50	3	0
		*Rhinolophus stheno*	4	0	0	0	0	0	0
		*Rhinolophus affinis*	3	0	0	0	0	0	0
		*Rhinolophus thomasi*	2	0	0	0	0	0	0
		*Rhinolophus pusillus*	1	0	0	0	0	0	0
		*Rhinolophus accumiatus*	1	0	0	0	0	0	0
		*Rhinolophus refulgens*	1	0	0	0	0	0	0
		*Rhinolophus* sp.	1	0	0	0	0	0	0
Vespertilionidae	*Myotis*	*Myotis siligorensis*	69	9	7	14	43.48	46	31
		*Myotis muricola*	3	0	0	0	0	0	0
	*Kerivoula*	*Kerivoula hardwickii*	1	0	0	0	0	0	0
	*Murina*	*Murina* sp.	1	0	0	0	0	0	0
Pteropodidae	*Cynopterus*	*Cynopterus brachyotis*	3	0	0	0	0	0	0
	*Eonycteris*	*Eonycteris spelaea*	2	0	0	0	0	0	0
Craseonycteridae	*Craseonycteris*	*Craseonycteris thonglongyai*	139	2	0	0	1.44	2	0
Megadermatidae	*Megaderma*	*Megaderma spasma*	63	2	0	0	1.58	3	0
Total			1042	273	**9**	16		737	36

^*^A single bat individual was identified as co-infested with both Streblidae and Nycteribiidae

### Bat fly infestation by sex, study site, and status of bat

**Fig. 3:**
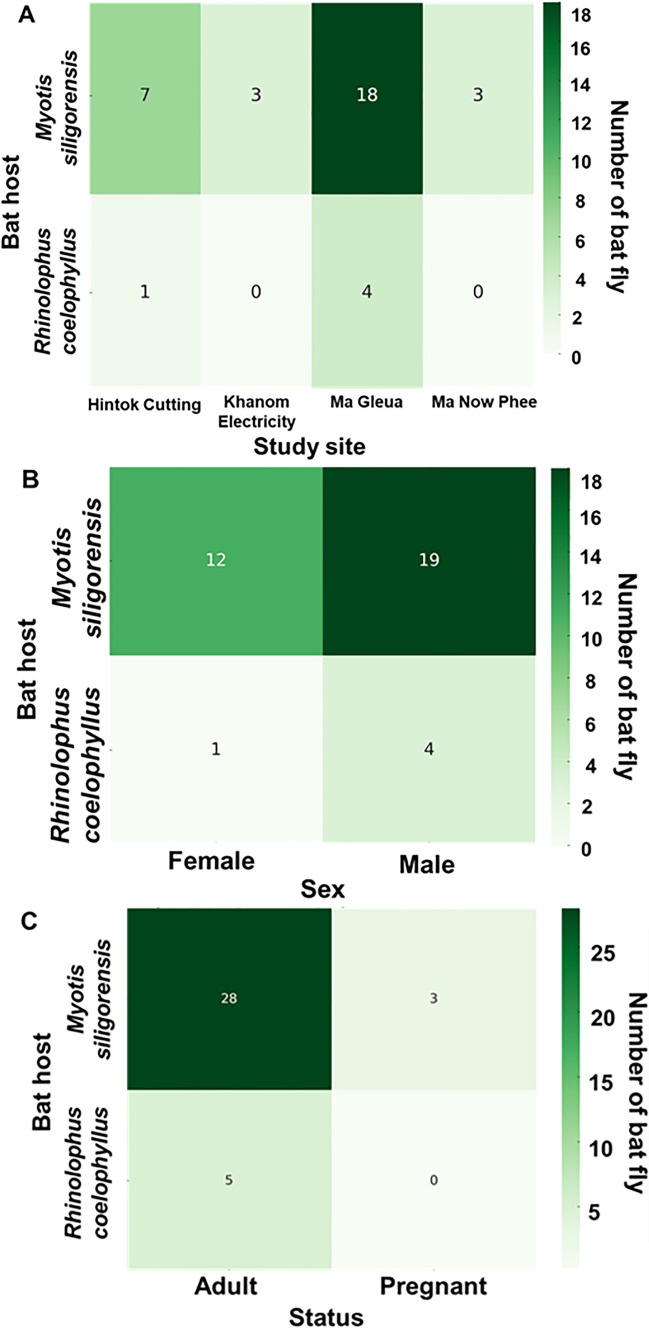
Distribution of host species infested by Streblidae across study sites (**A**), host sex (**B**), and reproductive status (**C**). The numbers inside the boxes in panels A–C indicate the number of bat flies. In panel C, the classification of adults by reproductive status includes all male and female bats except for pregnant females

The patterns of nycteribiid infestation across study sites, host sex, and physiological status are presented in Fig. 3 and Table S3. A total of 25 bats, representing two species (*Myotis siligorensis* and *Rhinolophus coelophyllus*), were infested with nycteribiid flies. The number of nycteribiids per bat ranged from one to four, with an average infestation rate of 1.44 nycteribiids per bat. The highest infestation rate was observed at Ma Gleua Cave, which accounted for 56% of the total infestations (14 out of 25 individuals), followed by Hintok Cutting (24%; 6 out of 25 individuals), Ma Now Phee Cave, and Khanom Electricity Generating Co., Ltd. (12% each, with three individuals from each site). Among the bat species, *My. siligorensis* hosted the majority of nycteribiids, accounting for 84% of all infestations (21 out of 25 individuals). Infestation patterns based on host sex revealed that male bats had higher infestation rates than females in both host species. Specifically, male *My. siligorensis* harbored 19 bat flies, whereas females had 12. Similarly, male *R. coelophyllus* carried four bat flies, while females carried only one. Additionally, 8% of the bats infested with nycteribiid flies were pregnant.

**Fig. 4:**
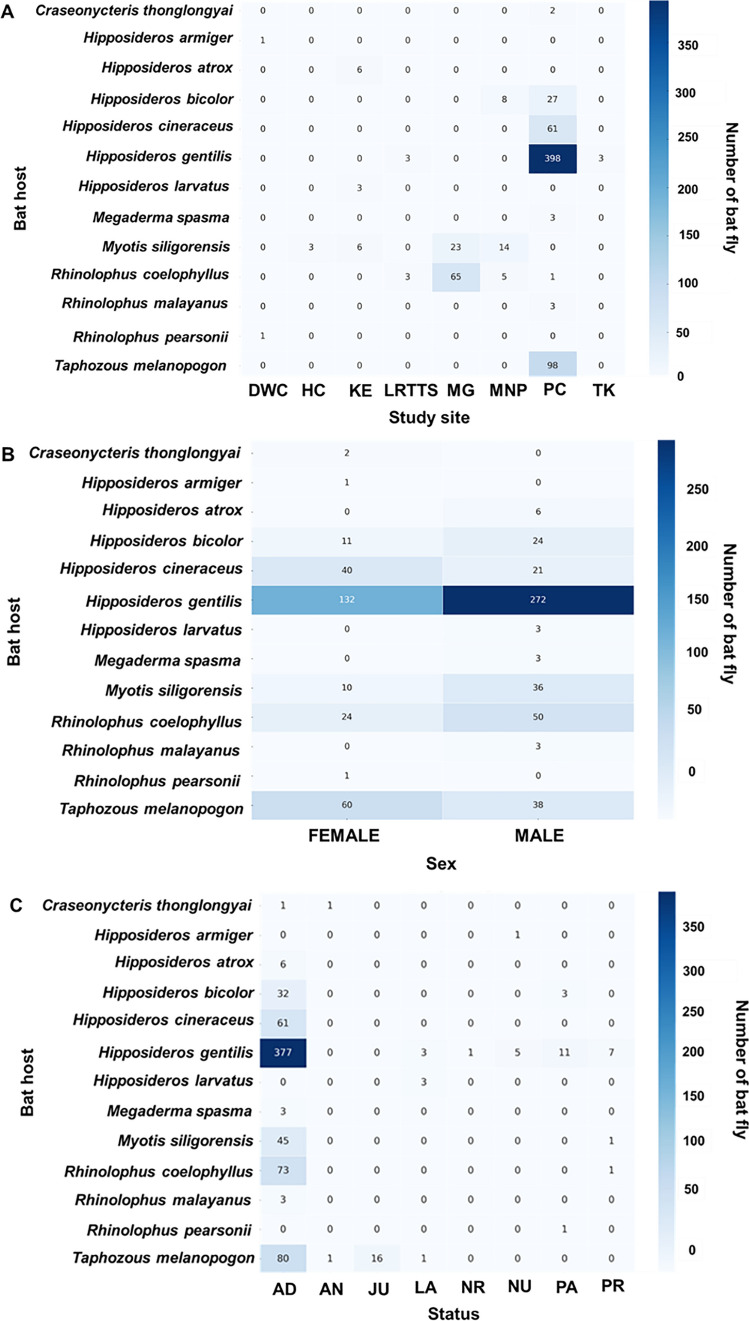
Distribution of host species infested by Streblidae across study sites (**A**), host sex (**B**), and reproductive status (**C**). *DWC* Daowadung Cave, *HC* Hintok Cutting, *KE* Khanom Electricity, *LRTSS* Lainan Research and Technology Transfer Station, *MG* Ma Gleua, *MNP* Ma Now Phee, *PC* Phra Cave, and *TC* Ta-Klor Cave. Bat reproductive status abbreviations: *AD* = adult, *AN* = adult nulliparous, *JU* = juvenile, *LA* = lactating, *NR* = nonreproductive, *NU* = nulliparous, *PA* = parous, and *PR* = pregnant

The distribution of Streblidae infestations was also examined across study sites, host sex, and physiological status, as presented in Fig. 4 and Table S3. A total of 289 bats, representing 13 species, were identified as hosts of Streblidae flies. The number of streblids per bat ranged from one to 28, with a mean infestation intensity of 2.55 streblids per bat. Infestation prevalence showed significant spatial variation, with the majority of infestations recorded at Phra Cave (74.4%; 215/289), followed by Ma Gleua and Ma Now Phee. Infestation prevalence also varied by host sex, with males exhibiting a significantly higher proportion of infestations (61.9%; 179/289) than females (38.1%; 110/289). Moreover, adult bats accounted for 87.5% of all recorded infestations (253/289), whereas juveniles represented only a minor fraction (3.1%; 9/289).

The Poisson generalized linear model (GLM) analysis revealed that bat species and physiological status significantly influenced bat fly infestation rates, whereas sex and study site did not show significant effects. Streblid infestation was notably higher in *Hipposideros larvatus* (*p* = 0.046). Additionally, pregnant bats had significantly lower infestation rates compared to non-pregnant individuals (*p* = 0.003). In the overall infestation model, *H. larvatus* remained significantly more infested than other bat species (*p* = 0.041), while pregnancy continued to be associated with reduced infestation levels (*p* = 0.006). In contrast, within the Nycteribiid infestation model, none of the tested predictors reached statistical significance, likely due to the limited sample size (*n* = 25). A detailed summary of the GLM results is provided in Table S4.

### Diversity of bat flies based on morphological and molecular identification

Through morphological identification, three genera of bat fly—*Raymondia*, *Brachytarsina*, and *Nycteribia*—were identified. Molecular analyses targeting mitochondrial (*Cox1*) and nuclear (28S rRNA) markers were conducted to evaluate species diversity. A total of 242 sequences (121 *Cox1* and 121 28S rRNA sequences) were generated from 121 specimens collected from bat hosts across various locations in Thailand. Using a species identification threshold of ≥ 97% similarity, eight *Cox1* sequences matched the *Brachytarsina kanoi* isolate from Japan (GenBank accession no. AB632571) with percent identities ranging from 98.14% to 98.31%, allowing for confident species-level classification. In contrast, the remaining 113 *Cox1* sequences demonstrated lower similarity to existing GenBank entries, and none of the 28S rRNA sequences showed matches with available GenBank records for these genera. Notably, this study represents the first genetic characterization of *Raymondia*, *Brachytarsina*, and *Nycteribia* using 28S rRNA sequences, thereby expanding molecular knowledge of these taxa.

### Phylogenetic analysis of bat fly Cox1 and 28S rRNA genes and species delimitation

**Fig. 5:**
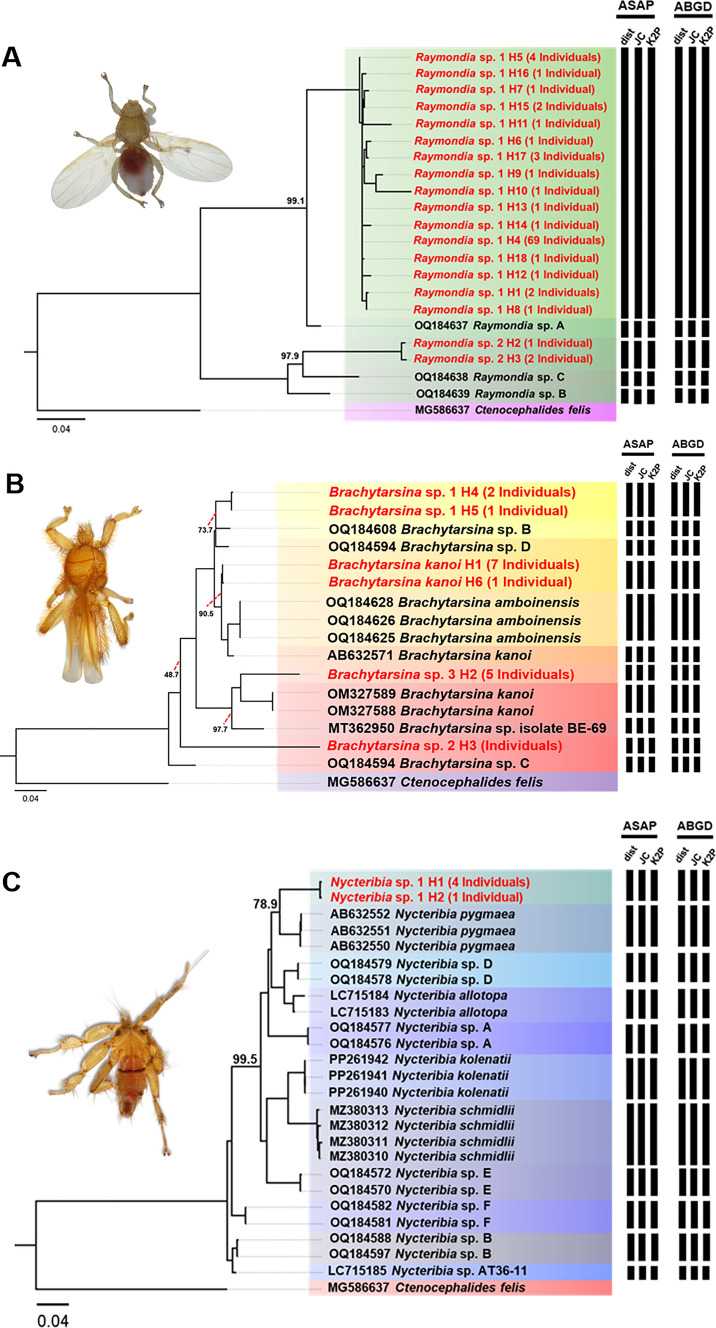
Phylogenetic trees of Raymondia (**A**), Brachytarsina (**B**), and Nycteribia (**C**) based on 157 nucleotide sequences of the Cox1 gene (538 bp), with Ctenocephalides felis (MG586637) used as outgroup inferred using the Maximum Likelihood (ML) method with the GTR + F + I + F4 model. Numbers near the branches indicate bootstrap values, representing the statistical support for the relationship between our sequence and the nearest reference sequence, as determined using ultrafast bootstrapping with 1000 replications. Additionally, species delimitation based on Cox1gene sequences (538 bp) was performed using the ASAP and ABGD web servers, incorporating three substitution models: simple distance (p-distance), Jukes–Cantor (JC69), and Kimura 2-Parameter (K2P). Black bars denote species delineated by each substitution model

In this phylogenetic analysis, a total of 121 cytochrome oxidase 1 (*Cox1*) sequences (each 538 bp) and 37 additional *Cox1* sequences retrieved from the GenBank database were used. The *Cox1* sequence of *Ctenocephalides felis* (MG586637) served as an outgroup. Phylogenetic analysis revealed distinct clades representing various genera and species of bat flies, offering valuable insights into their evolutionary relationships (Fig. 5). Detailed sequence data, including accession numbers, percentage identity, and query coverage, are provided in Supplementary Table S5. A major clade consisting of 92 individuals exhibited 99.1% bootstrap support and was identified as *Raymondia* sp. 1, which shared 96.9% sequence similarity with the reference sequence *Raymondia* sp. A (OQ184637). Similarly, three individuals formed a distinct clade, identified as *Raymondia* sp. 2, which also received 100% bootstrap support and showed a 97.9% sequence similarity with the reference sequence *Raymondia* sp. C (OQ184638). The genus *Brachytarsina* demonstrated significant phylogenetic complexity, forming four distinct clades, each corresponding to a separate species. The first clade, identified as *B. kanoi*, showed a BLASTN identity exceeding 98% and a bootstrap value of 90.5% relative to the reference sequence *B. kanoi* (AB632571). The second clade, comprising three individuals identified as *Brachytarsina sp.* 1, demonstrated strong bootstrap support (99.9%) but exhibited lower similarity (73.7%) to the reference sequences *Brachytarsina* sp. B and *Brachytarsina* sp. D (OQ184608 and OQ184594, respectively) from Hong Kong. The third clade, consisting of five individuals identified as *Brachytarsina* sp. 2, received 100% bootstrap support, although it displayed low sequence similarity (56%) with the reference sequence *Brachytarsina* sp. C (OQ184589). The fourth clade, comprising five individuals identified as *Brachytarsina* sp. 3, exhibited low bootstrap support compared to the reference sequences of *B. kanoi* (OM327589 and OM327588) from Pakistan and *Brachytarsina* sp. isolate BE-69 (MT362950) from Korea. Within the genus *Nycteribia*, seven clades were identified. Five sequences from this study formed a distinct clade with robust bootstrap support (100%) and were identified as *Nycteribia* sp. 1. This clade was phylogenetically distinct from the six clades of the reference sequences, which exhibited comparatively lower bootstrap support. Species delimitation was conducted using the Assemble Species by Automatic Partitioning (ASAP) and Automatic Barcode Gap Discovery (ABGD) algorithms. Both approaches consistently identified seven distinct hypothetical species, including two species of *Raymondia*, four species of *Brachytarsina*, and one species of *Nycteribia* (Fig. 5). These findings align with the phylogenetic tree reconstructed using the same gene, which is further corroborated by high bootstrap support values, providing robust validation for the proposed species classifications.

**Fig. 6:**
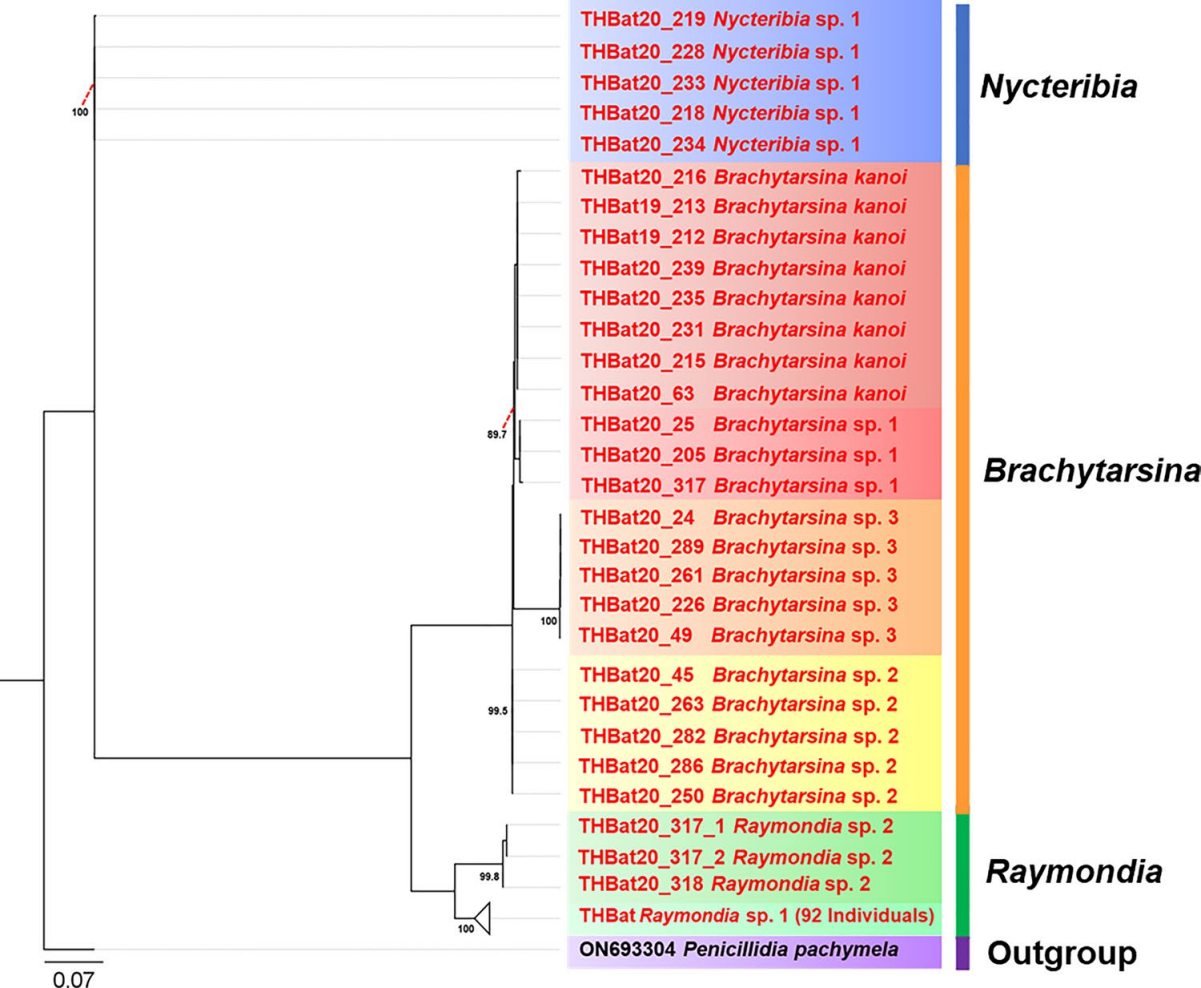
Phylogenetic tree of bat flies based on 121 nucleotide sequences of the 28S rRNA gene (497 bp), with *Penicillidia pachymela* (ON693304) serving as the outgroup. The tree was inferred using the maximum likelihood (ML) method with the F81 + F + G4 model. Bootstrap values, calculated using ultrafast bootstrapping with 1000 replications, are shown at the corresponding branches

The phylogenetic tree based on 28S rRNA was reconstructed using 121 sequences (498 bp), with *Penicillidia pachymela* (ON693304) serving as the outgroup, employing the maximum likelihood (ML) method. Due to the unavailability of 28S rRNA sequence data for the three genera identified in the study, representatives from these genera were excluded from the ingroup in the phylogenetic reconstruction. The resulting 28S rRNA tree closely mirrored the topology of the *Cox1*-based phylogeny, delineating seven well-supported and distinct clades (Fig. 6).

### Haplotype network analysis based on the Cox1 sequences of bat fly species

**Fig. 7:**
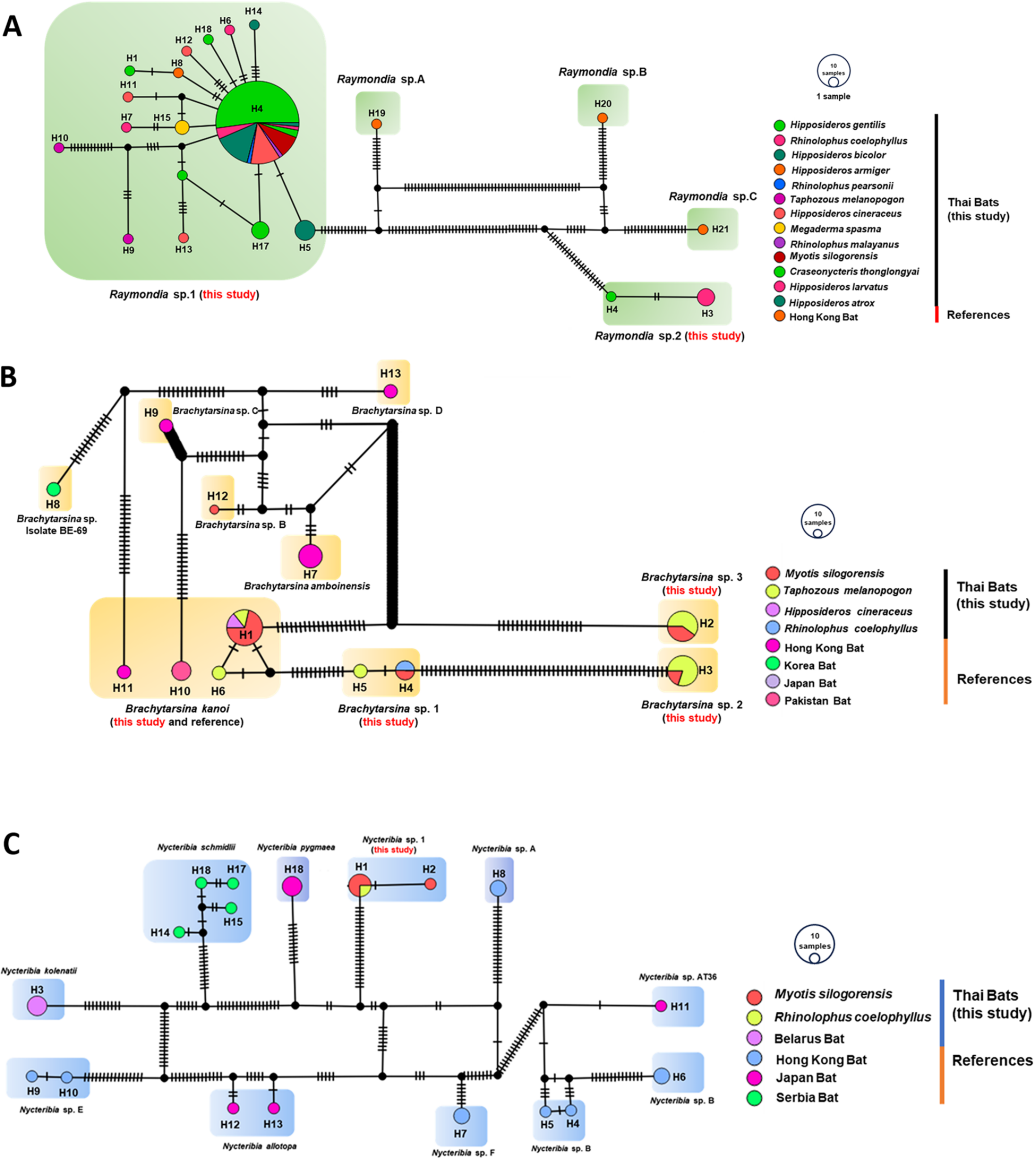
TCS network of partial *Cox1* gene sequences from *Raymondia* (**A**), *Brachytarsina* (**B**), and *Nycteribia* (**C**), including reference data from GenBank. Each circle represents a haplotype, with branch markings indicating the number of mutations. Colors correspond to the host species or geographic origin. The size of each node represents the frequency or number of samples for each haplotype. Black circles denote missing haplotypes or median vectors

The study's findings, as detailed in Table 2, provide a comprehensive summary of the diversity of bat fly species and their haplotypes across various study sites and bat hosts. Complementing this, Fig. 7 visually illustrates the distribution of haplotypes among hosts, offering insights into patterns of host specificity and genetic diversity. A total of 18 haplotypes were identified within the genus *Raymondia*, including 16 haplotypes for *Raymondia* sp. 1 (H1, H4–H18) and two haplotypes for *Raymondia* sp. 2 (H2, H3). Additionally, six haplotypes were recorded in the genus *Brachytarsina*, encompassing *Brachytarsina kanoi* (H1, H6), *Brachytarsina* sp. 1 (H4, H5), *Brachytarsina* sp. 2 (H3), and *Brachytarsina* sp. 3 (H2). The genus *Nycteribia* comprised two haplotypes. The distribution of *Raymondia* sp. 1 and its associated haplotypes varied substantially across the study sites. At Daowadung Cave, *Hipposideros armiger* and *Rhinolophus pearsonii* hosted *Raymondia* sp. 1, with haplotypes H8 and H4, respectively. *Raymondia* sp. 1 with haplotype H4 was also detected at Hintok Cutting and Khanom Electricity, hosted by *Myotis siligorensis* and *Hipposideros* species. At the Lainan Research and Technology Transfer Station, *Hipposideros gentilis* hosted *Raymondia* sp. 1 (H1), while *Rhinolophus coelophyllus* harbored *Raymondia* sp. 2 (H2, H3), and *Taphozous melanopogon* was associated with *Brachytarsina* sp. 1 (H5).

**Table 2:** Overview of bat fly species diversity and haplotype distribution across study sites and bat hosts

Study site	Bat host	Bat fly species	Haplotypes with count
DC	*Hipposideros armiger*	*Raymondia* sp. 1	H8 (1)
	*Rhinolophus pearsonii*	*Raymondia* sp. 1	H4 (1)
HC	*Myotis siligorensis*	*Raymondia* sp. 1	H4 (3)
KE	*Hipposideros atrox*	*Raymondia* sp. 1	H4 (1)
	*Hipposideros larvatus*	*Raymondia* sp. 1	H4 (1)
	*Myotis siligorensis*	*Raymondia* sp. 1	H4 (2)
LRTTS	*Hipposideros gentilis*	*Raymondia* sp. 1	H1 (3),
	*Rhinolophus coelophyllus*	*Raymondia* sp. 2	H2 (1), H3 (2)
	*Taphozous melanopogon*	*Brachytarsina* sp. 1	H5 (1)
MG	*Myotis siligorensis*	*Brachytarsina kanoi*	H1 (3)
	*Myotis siligorensis*	*Brachytarsina* sp. 1	H4 (1)
	*Myotis siligorensis*	*Brachytarsina* sp. 3	H2 (1)
	*Myotis siligorensis*	*Nycteribia* sp. 1	H1 (2), H2 (1)
	*Rhinolophus coelophyllus*	*Nycteribia* sp. 1	H1 (2)
	*Rhinolophus coelophyllus*	*Raymondia* sp. 1	H4 (3), H6 (1), H7 (1)
	*Taphozous melanopogon*	*Brachytarsina kanoi*	H1 (1), H6 (1)
MNP	*Hipposideros bicolor*	*Raymondia* sp. 1	H4 (5), H5 (4)
MNP	*Myotis siligorensis*	*Brachytarsina kanoi*	H1 (2)
PC	*Craseonycteris thonglongyai*	*Raymondia* sp. 1	H4 (2)
	*Hipposideros bicolor*	*Raymondia* sp. 1	H14 (1), H4 (5)
	*Hipposideros cineraceus*	*Brachytarsina kanoi*	H1 (1)
	*Hipposideros cineraceus*	*Raymondia* sp. 1	H11 (1), H12 (1), H4 (9)
	*Hipposideros gentilis*	*Raymondia* sp. 1	H13 (1), H16 (1), H17 (3), H18 (1), H4 (32)
	*Megaderma spasma*	*Raymondia* sp. 1	H15 (2)
	*Myotis siligorensis*	*Brachytarsina* sp. 2	H3 (1)
	*Myotis siligorensis*	*Brachytarsina* sp. 3	H2 (1)
	*Myotis siligorensis*	*Raymondia* sp. 1	H4 (1)
	*Rhinolophus coelophyllus*	*Brachytarsina* sp. 1	H4 (1)
	*Rhinolophus malayanus*	*Raymondia* sp. 1	H4 (1)
	*Taphozous melanopogon*	*Brachytarsina* sp. 2	H3 (4)
	*Taphozous melanopogon*	*Brachytarsina* sp. 3	H2 (3)
	*Taphozous melanopogon*	*Raymondia* sp. 1	H10 (1), H9 (1)
TC	*Hipposideros gentilis*	*Raymondia* sp. 1	H4 (3)

*DWC* Daowadung Cave, *HC* Hintok Cutting, *KE* Khanom Electricity, *LRTSS* Lainan Research and Technology Transfer Station, *MG* Ma Gleua, *MNP* Ma Now Phee, *PC* Phra Cave, and *TC* Ta-Klor Cave

The Ma Gleua site exhibited greater diversity, with *My. siligorensis* hosting *Brachytarsina kanoi* (H1), *Brachytarsina* sp. 1 (H4), and *Brachytarsina* sp. 3 (H2). *Rhinolophus coelophyllus* hosted *Raymondia* sp. 1 (H4, H6, H7) and *Nycteribia* sp. 1 (H1, H2). At Ma Now Phee, *Hipposideros bicolor* supported *Raymondia* sp. 1 (H4, H5), while Phra Cave exhibited a high frequency of haplotype H4 in 32 individuals of *H. gentilis*, alongside other haplotypes (H13–H18) in the same host species. At Ta-Klor Cave, *H. gentilis* also hosted *Raymondia* sp. 1 (H4). These findings underscore the widespread occurrence of *Raymondia* sp. 1 across a range of bat species and study sites. Notably, haplotype H4 emerged as the most prevalent, suggesting its significant role in shaping the genetic structure of *Raymondia* sp. 1 populations. The pervasive distribution of haplotype H4 likely reflects ecological factors, such as host availability, and possible local adaptations to specific environmental conditions, highlighting the intricate dynamics among host species, parasites, and their environments.

### Tripatrid network analysis

**Fig. 8:**
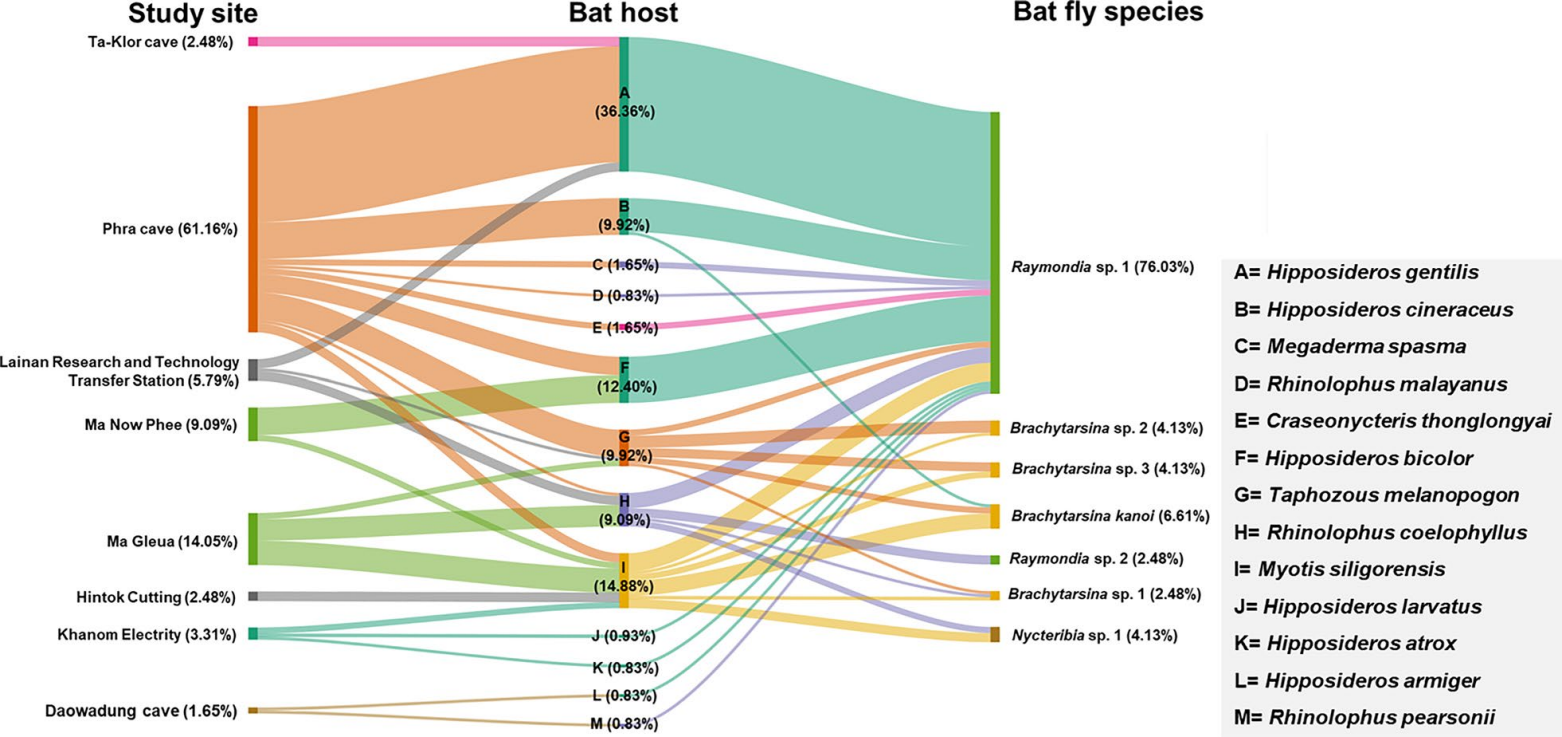
Tripartite network analysis based on study site, bat host, and bat fly species. The percentage values represent the relative abundance of the 121 bat fly specimens used in this analysis

The tripartite network analysis, incorporating study sites, bat hosts, and bat fly species, is illustrated in Fig. 8. The analysis identified *Raymondia* sp. 1 as the most prevalent bat fly species, widely distributed across multiple study sites and bat hosts. Notably, 76.03% (92/121) of *Raymondia* sp. 1 individuals were recorded infesting various bat species, with a significant proportion (31.40%; 38/121) infesting *Hipposideros gentilis* at Phra Cave. In contrast, *Raymondia* sp. 2 was exclusively found infesting *Rhinolophus coelophyllus* at the Lainan Research and Technology Transfer Station, accounting for 2.48% (3/121) of all bat fly individuals.

The genus *Brachytarsina*, comprising *Brachytarsina kanoi* and three unidentified species (*Brachytarsina* sp. 1, sp. 2, and sp. 3), displayed a broader distribution across different sites and bat hosts. *Brachytarsina kanoi* was identified in three individuals of *Myotis siligorensis* (2.48%, *n* = 121) and two individuals of *Taphozous melanopogon* (1.65%, *n* = 121) at Ma Gleua Cave. *Brachytarsina* sp. 1 was observed in one *My. siligorensis* individual at Ma Gleua Cave (0.83%, *n* = 121) and one *R. coelophyllus* individual at Phra Cave Cave (0.83%, *n* = 121). *Brachytarsina* sp. 2 was found in one *My. siligorensis* individual at Phra Cave (0.83%, *n* = 121), while *Brachytarsina* sp. 3 infested one *My. siligorensis* individual at Ma Gleua Cave (0.83%, *n* = 121) and three *T. melanopogon* individuals at Phra Cave (2.48%, *n* = 121). Similarly, *Nycteribia* sp. 1 was detected in three *My. siligorensis* individuals (2.48%) and two *R. coelophyllus* individuals (1.65%) at Ma Gleua Cave. These findings highlight the diversity and host associations of bat fly species across the study sites, with *Raymondia* sp. 1 emerging as the dominant species and exhibiting the highest prevalence among bat hosts.

### Genetic variations and estimates of intra- and interspecific sequence divergences of Cox1 and 28S rRNA sequences of bat flies in Thailand

The genetic diversity of *Cox1* and 28S rRNA sequences among the seven bat fly species collected in Thailand revealed substantial interspecific and intraspecific variations (Table 3). *Raymondia* sp. 1 exhibited the highest genetic diversity across both markers. The *Cox1* gene displayed 111 polymorphic sites and 18 haplotypes, resulting in a haplotype diversity (Hd) of 0.4371 ± 0.066 and a nucleotide diversity (π) of 0.0060 ± 0.04800. Similar trends were observed for the 28S rRNA gene, where *Raymondia* sp. 1 exhibited 35 polymorphic sites and 21 haplotypes, with Hd and π values of 0.765 ± 0.034 and 0.00523 ± 0.01485, respectively. Statistically significant negative values of Tajima’s D (– 2.88120) and Fu and Li’s D (– 8.19923) for the *Cox1* gene suggest either population expansion or purifying selection for this gene. In contrast, *Brachytarsina* sp. 2 and 3 exhibited no genetic variation across either the *Cox1* or 28S rRNA markers, with single haplotype and nucleotide diversity values of π = 0.00000. This lack of diversity suggests a potential bottleneck or the presence of a recently established population. Moderate genetic diversity was observed in other species, such as *B. kanoi* and *Nycteribia* sp. 1. *Brachytarsina kanoi* displayed low haplotype diversity (Hd = 0.250 ± 0.180) and nucleotide diversity (π = 0.00047 ± 0.0007) for the *Cox1* gene, with similar trends for the 28S rRNA marker. *Nycteribia* sp. 1 showed higher haplotype diversity (Hd = 0.400 ± 0.237) and nucleotide diversity (π = 0.00082 ± 0.0009 in the *Cox1* gene), indicating greater genetic variation within this species. Neutrality tests for both species did not yield significant results, suggesting that their evolution follows a neutral model.

**Table 3:** Genetic diversity indices and neutrality test of *Cox1* and *28S rRNA* sequences from seven species of bat flies in Thailand

Species	Marker	N	Site (bp)	S	h	Hd ± SD	π ± SD	Tajima’s D	Fu and Li’s D
*Raymondia* sp. 1	*Cox1*	92	539	111	18	0.437 ± 0.066	0.00660 ± 0.04800	−2,88,120*	−8,19,923*
	28 s rRNA	92	497	35	21	0.765 ± 0.034	0.00523 ± 0.01485	−2,03074*	−2.11429
*Raymondia* sp. 2	*Cox1*	3	598	5	2	0.667 ± 0.314	0.00563 ± 0.00546	NA	NA
	28 s rRNA	3	497	3	2	0.667 ± 0.314	0.00273 ± 0.00273	NA	NA
*Brachytarsina kanoi*	*Cox1*	8	528	1	2	0.250 ± 0.180	0.00047 ± 0.00073	−1.05482	−1. 12,639
	28 s rRNA	8	497	2	2	0.250 ± 0.180	0.00102 ± 0.00158	−1.31009	−1.40980
*Brachytarsina* sp. 1	*Cox1*	3	528	1	2	0.667 ± 0.314	0.00126 ± 0.00126	NA	NA
	28 s rRNA	3	497	2	2	0.667 ± 0.314	0.00273 ± 0.00273	NA	NA
*Brachytarsina* sp. 2	*Cox1*	5	528	0	1	0.000 ± 0.000	0.00000 ± 0.000	NA	NA
	28 s rRNA	5	497	0	1	0.000 ± 0.000	0.00000 ± 0.000	NA	NA
*Brachytarsina* sp. 3	*Cox1*	5	598	1	1	0.000 ± 0.000	0.00000 ± 0.000	NA	NA
	28 s rRNA	5	497	2	2	0.400 ± 0.237	0.00164 ± 0.00196	−0.97256	−0.97256
*Nycteribia* sp. 1	*Cox1*	5	518	1	2	0.400 ± 0.237	0.00077 ± 0.00093	−0.81650	−0.81650
	28 s rRNA	5	497	1	2	0.400 ± 0.237	0.00082 ± 0.00098	−0.81650	−0.81650

The analysis of the *Cox1* gene revealed intraspecific sequence divergence ranging from 0.00 to 0.01, with *Raymondia* sp. 1 exhibiting the highest values and *Brachytarsina* sp. 2 showing no divergence. Interspecific divergence was notably higher, ranging from 0.09 to 0.33, with the largest divergence observed between *Raymondia* sp. 2 and *Nycteribia* sp. 1 as well as between *Brachytarsina* sp. 3 and *Nycteribia* sp. 1 (0.33). Within *Brachytarsina*, interspecific divergence ranged from 0.10 to 0.19, reflecting moderate genetic differentiation. The divergence between *Nycteribia* and other genera was significantly higher (0.25 – 0.30), underscoring clear phylogenetic separation (Table 4).

**Table 4:** Estimates of intra- and interspecific sequence divergences as measured by the *Cox1*

	1	2	3	4	5	6	7	8	9	10	11	12	13	14	15	16	17	18	19	20	21
THBat20_314_1 *Raymondia* sp. 1																					
THBat20_300 *Raymondia* sp. 1	0.01																				
THBat22_58 *Raymondia* sp. 1	0.01	0.00																			
THBat20_317_1 *Raymondia* sp. 2	0.22	0.22	0.22																		
THBat20_317_2 *Raymondia* sp. 2	0.22	0.22	0.22	0.00																	
THBat20_318 *Raymondia* sp. 2	0.22	0.22	0.22	0.00	0.00																
THBat20_239 *Brachytarsina kanoi*	0.26	0.25	0.26	0.23	0.23	0.23															
THBat19_212 *Brachytarsina kanoi*	0.26	0.25	0.26	0.23	0.23	0.23	0.00														
THBat20_63 *Brachytarsina kanoi*	0.26	0.25	0.26	0.23	0.23	0.23	0.00	0.00													
THBat20_205 *Brachytarsina* sp. 1	0.27	0.26	0.26	0.23	0.23	0.23	0.03	0.03	0.03												
THBat20_317 *Brachytarsina* sp. 1	0.26	0.26	0.26	0.24	0.24	0.24	0.03	0.03	0.03	0.00											
THBat20_225 *Brachytarsina* sp. 1	0.27	0.26	0.26	0.23	0.23	0.23	0.03	0.03	0.03	0.00	0.00										
THBat20_45 *Brachytarsina* sp. 2	0.29	0.29	0.28	0.32	0.32	0.32	0.17	0.17	0.17	0.16	0.16	0.16									
THBat20_50 *Brachytarsina* sp. 2	0.29	0.29	0.28	0.32	0.32	0.32	0.17	0.17	0.17	0.16	0.16	0.16	0.00								
THBat20_286 *Brachytarsina* sp. 2	0.29	0.29	0.28	0.32	0.32	0.32	0.17	0.17	0.17	0.16	0.16	0.16	0.00	0.00							
THBat20_289 *Brachytarsina* sp. 3	0.27	0.27	0.27	0.25	0.25	0.25	0.10	0.10	0.10	0.11	0.11	0.11	0.20	0.20	0.20						
THBat20_226 *Brachytarsina* sp. 3	0.27	0.27	0.27	0.25	0.25	0.25	0.10	0.10	0.10	0.11	0.11	0.11	0.20	0.20	0.20	0.00					
THBat20_49 *Brachytarsina* sp. 3	0.27	0.27	0.27	0.25	0.25	0.25	0.10	0.10	0.10	0.11	0.11	0.11	0.20	0.20	0.20	0.00	0.00				
THBat20_218 *Nycteribia* sp. 1	0.32	0.31	0.31	0.34	0.34	0.34	0.28	0.28	0.28	0.28	0.28	0.28	0.29	0.29	0.29	0.33	0.33	0.33			
THBat20_233 *Nycteribia* sp. 1	0.32	0.31	0.31	0.34	0.34	0.34	0.28	0.28	0.28	0.28	0.28	0.28	0.29	0.29	0.29	0.33	0.33	0.33	0.00		
THBat20_234 *Nycteribia* sp. 1	0.32	0.31	0.30	0.34	0.34	0.34	0.28	0.28	0.28	0.28	0.28	0.28	0.28	0.28	0.28	0.33	0.33	0.33	0.00	0.00	

Intraspecific sequence divergences are highlighted in yellow, whereas interspecific sequence divergences remain unhighlighted

Analysis of intra- and interspecific sequence divergences based on the 28S rRNA gene revealed distinct patterns among the studied bat fly species (Table 5). Intraspecific divergences ranged from 0.00 to 0.01 within most *Raymondia* species, demonstrating a high degree of genetic homogeneity. Specifically, *Raymondia* sp. 1 exhibited negligible divergence among samples (0.00 – 0.01), suggesting minimal genetic differentiation within the species. Similarly, *B. kanoi* showed no intraspecific divergence across all samples, indicating genetic stability. In contrast, interspecific divergences among *Raymondia* species and between *Raymondia* and other genera were significantly higher. *Raymondia* sp. 2 displayed an average interspecific divergence of 0.21 compared to *Raymondia* sp. 1, indicating a substantial genetic separation between these two species. Among the *Brachytarsina* species, *Brachytarsina* sp. 1, *B.* sp. 2, and *B.* sp. 3 exhibited higher interspecific divergence values (0.00 – 0.29), reflecting their genetic distinctiveness despite being in the same genus. Notably, *Nycteribia* sp. 1 displayed the highest intraspecific divergence (0.00–0.01) while maintaining substantial interspecific divergence compared to other genera. For example, *Nycteribia* sp. 1 diverged from *Brachytarsina kanoi* and *Raymondia* sp. 1 by 0.32 and 0.33, respectively, underscoring its unique genetic identity.

**Table 5:** Estimates of intra- and interspecific sequence divergences as measured by the 28S rRNA

	1	2	3	4	5	6	7	8	9	10	11	12	13	14	15	16	17	18	19	20	21
THBat20_314 *Raymondia* sp. 1																					
THBat20_300 *Raymondia* sp. 1	0.01																				
THBat22_58 *Raymondia* sp. 1	0.01	0.01																			
THBat20_317_1 *Raymondia* sp. 2	0.09	0.09	0.08																		
THBat20_317_2 *Raymondia* sp. 2	0.09	0.09	0.08	0.00																	
THBat20_318 *Raymondia* sp. 2	0.08	0.09	0.08	0.00	0.00																
THBat20_239 *Brachytarsina kanoi*	0.19	0.19	0.20	0.23	0.23	0.23															
THBat19_212 *Brachytarsina kanoi*	0.19	0.19	0.20	0.23	0.23	0.23	0.00														
THBat20_63 *Brachytarsina kanoi*	0.19	0.19	0.20	0.23	0.23	0.23	0.00	0.00													
THBat20_205 *Brachytarsina* sp. 1	0.20	0.20	0.20	0.24	0.24	0.23	0.01	0.01	0.01												
THBat20_317 *Brachytarsina* sp. 1	0.20	0.20	0.21	0.24	0.24	0.24	0.01	0.02	0.01	0.00											
THBat20_225 *Brachytarsina* sp. 1	0.20	0.20	0.20	0.24	0.24	0.23	0.01	0.01	0.01	0.00	0.00										
THBat20_45 *Brachytarsina* sp. 2	0.19	0.19	0.19	0.23	0.23	0.23	0.00	0.01	0.00	0.01	0.01	0.01									
THBat20_50 *Brachytarsina* sp. 2	0.19	0.19	0.19	0.23	0.23	0.23	0.00	0.01	0.00	0.01	0.01	0.01	0.00								
THBat20_286 *Brachytarsina* sp. 2	0.19	0.19	0.19	0.23	0.23	0.23	0.00	0.01	0.00	0.01	0.01	0.01	0.00	0.00							
THBat20_289 *Brachytarsina* sp. 3	0.26	0.26	0.26	0.29	0.29	0.29	0.06	0.06	0.06	0.06	0.07	0.06	0.06	0.06	0.06						
THBat20_226 *Brachytarsina* sp. 3	0.26	0.26	0.26	0.29	0.29	0.29	0.06	0.06	0.06	0.06	0.07	0.06	0.06	0.06	0.06	0.00					
THBat20_49 *Brachytarsina* sp. 3	0.26	0.26	0.26	0.29	0.29	0.29	0.06	0.06	0.06	0.06	0.07	0.06	0.06	0.06	0.06	0.00	0.00				
THBat20_218 *Nycteribia* sp. 1	0.43	0.41	0.43	0.43	0.43	0.42	0.48	0.49	0.48	0.49	0.50	0.49	0.48	0.48	0.48	0.53	0.53	0.53			
THBat20_233 *Nycteribia* sp. 1	0.43	0.41	0.43	0.43	0.43	0.42	0.48	0.49	0.48	0.49	0.50	0.49	0.48	0.48	0.48	0.53	0.53	0.53	0.00		
THBat20_234 *Nycteribia*_sp. 1	0.43	0.42	0.43	0.43	0.43	0.43	0.48	0.49	0.48	0.49	0.50	0.49	0.48	0.48	0.48	0.53	0.53	0.53	0.00	0.00	

Intraspecific sequence divergences are highlighted in yellow, whereas interspecific sequence divergences remain unhighlighted

## Discussion

Research on bat flies and the pathogens they transmit in Thailand remains limited, resulting in significant knowledge gaps regarding their ecological roles and public health implications. Building on the foundational work of Samoh et al. [5] and Hill and McNeely [35], who provided a morphology-based checklist of Nycteribiidae and Streblidae bat flies in the region, this study seeks to expand the understanding of bat fly diversity and host-parasite dynamics. Employing a multifaceted approach that integrates morphological identification, molecular analysis, and ecological surveys, this investigation provides a comprehensive representation of bat fly diversity and distribution.

The prevalence of bat fly infestations varied among the sampling sites, with host-parasite interactions examined across eight locations. The findings revealed a greater diversity of Streblidae compared to Nycteribiidae species infesting Thai bats. Notably, Nycteribiidae infestations were exclusively observed in *My. siligorensis* and *R. coelophyllus,* which was not unexpected given the bat diversity and the number of individuals trapped in this study. This contrasts with the general biogeographic pattern where Nycteribiidae species are predominantly associated with Old World bats [7, 17]. The overall infestation rate (28.59%) was significantly lower than the rates reported in Korea (74%) and Mexico (68%) [36, 37]. These differences may reflect variations in bat population density, roosting ecology, and environmental conditions, which play critical roles in shaping host-parasite dynamics and bat fly distribution [20, 38–40]. Additionally, infestation rates should be interpreted with caution, as they may be influenced by sampling protocols. Because some bat flies, particularly winged species, are temporary ectoparasites, bats may be classified as uninfested if no flies were collected at the time of sampling, even if they had previously hosted parasites. Furthermore, infestation intensity was positively correlated with bat population size, suggesting that denser populations facilitate greater parasite burdens through increased host contact and ectoparasite transmission [41].

Host quality, defined as the suitability of a bat host to support ectoparasites, is influenced by various factors such as age, sex, physiological condition, health status, behavior, and body size [42–44]. These factors significantly shape infestation patterns. Male bats exhibited higher levels of Streblidae and Nycteribiidae infestations compared to females, consistent with studies on mammalian ectoparasites, which often report greater parasitism in males. This phenomenon is typically attributed to lower immune competence in males, influenced by the immunosuppressive effects of testosterone [45–48]. However, this finding contrasts with certain other studies on bats, where females are observed to experience higher infestation rates [48–50]. Gravid and lactating females, potentially undergoing immunosuppression during reproduction, were also found to be more susceptible to parasitism. This increased vulnerability may negatively affect their health and reproductive fitness by raising physiological stress and depleting energy reserves during critical stages of reproduction [51–54]. Contrary to prevailing expectations, our generalized linear model (GLM) analysis demonstrated that pregnant bats exhibited significantly lower levels of bat fly infestation than their non-pregnant counterparts. This pattern aligns with observations in other ectoparasites; for instance*, Spinturnix emarginata* has been reported to have lower infestation rates in pregnant *Myotis emarginatus* [55].

This study employed an integrative approach, combining morphological analysis and genetic characterization to classify bat flies. Morphological analysis identified three genera, while molecular data delineated seven species. For instance, *Raymondia* sp. 1 shared morphological features with *Raymondia pagodarum*, including a distinct mesonotal suture, a unique calypteron shape, and the curvature of the fourth longitudinal wing vein [55]. This research also extended the geographic scope of bat fly studies in Thailand, incorporating specimens from Nan and Nakhon Si Thammarat provinces, previously unexamined regions. Furthermore, this study contributes new insights into bat flies and bat hosts in Kanchanaburi Province, building on previous research that exclusively documented infestations of *Phthiridium aff. szechuanum* on *Rhinolophus macrotis* bats [5]. Notably, this is the first study in Thailand to employ DNA barcoding for bat flies, utilizing mitochondrial *Cox1* and nuclear 28S rRNA markers.

The host specificity of ectoparasites is influenced by the behavioral, ecological, and evolutionary characteristics of their hosts, with roosting behavior being particularly significant [20, 56, 57]. Bats that occupy stable roosts, such as caves, often experience higher parasitism rates and harbor more diverse bat fly communities. Phylogenetic analysis based on *Cox1* revealed that *Raymondia* sp. 1 is polyxenous, parasitizing 12 bat species across five genera (*Hipposideros*, *Rhinolophus*, *Myotis*, *Megaderma*, and *Craseonycteris*). Notably, these bat hosts were found to be infected with a bat fly-borne blood protozoan, *Polychromophilus* [24, 26]. However, the vectorial competence of bat flies was not investigated in the current study. This finding contrasts with *Raymondia* sp. A from Hong Kong, which is monoxenous, parasitizing only *Hipposideros gentilis*. Similarly, *Raymondia* sp. 2 exhibited host specificity, parasitizing *R. coelophyllus*, and was closely related to *Raymondia* sp. C from Hong Kong. Instances of monoxenous bat flies on non-primary hosts may reflect transient associations that occur during host-roost switching.

Phylogenetic analyses also identified four distinct clades of *Brachytarsina*, with one species, *Brachytarsina kanoi*, identified based on high sequence similarity and bootstrap support. This species was associated with *My. siligorensis* and *T. melanopogon*, marking its first documentation in Thailand. In Korea, it has been reported on *Rhinolophus ferrumequinum* [36]. *Nycteribia* sp. 1 exhibited a 93.75–94.05% identity to *Nycteribia allotopa* but formed a distinct clade in the phylogenetic tree. This species was associated with *My. siligorensis* and *R. coelophyllus*, whereas *N. allotopa* has been previously reported in association with *Miniopterus* bats in other regions, including Hong Kong, Korea, and Japan [17, 58–61].

One limitation of this study was the uneven distribution of samples across locations, with a notable concentration in Kanchanaburi Province. Additionally, the limited availability of identification keys and reference sequences for genera such as *Raymondia* and *Brachytarsina* in the Barcode of Life Database (BOLD) and GenBank hindered species-level identification. Despite this, the *Cox1* and 28S rRNA markers proved effective for species differentiation, as supported by species delimitation analyses. The 28S rRNA gene sequences generated in this study represent the first molecular data for the genera *Raymondia*, *Brachytarsina*, and *Nycteribia*, significantly enhancing the molecular resources available for these taxa. These findings underscore the importance of integrating molecular tools to advance taxonomic and phylogenetic research, particularly for underexplored regions and taxa.

## Conclusions

This study represents the first molecular characterization of bat fly diversity in Thailand, providing critical insights into their genetic complexity, taxonomy, host specificity, and ecological interactions. The findings underscore the prevalence of bat fly infestations across a range of bat species, with Streblidae demonstrating greater species diversity than Nycteribiidae. Through the integration of morphological and molecular approaches, the study identifies distinct clades and haplotypes within the genera *Raymondia*, *Brachytarsina*, and *Nycteribia*, uncovering significant genetic diversity and intricate host-parasite relationships. The results enhance understanding of bat fly biology and their potential roles in host-vector-pathogen dynamics, laying a robust foundation for future research into their ecological significance and zoonotic implications.

## Supplementary Information

Additional file 1. Table S1. Oligonucleotide primers and PCR conditions used for the amplification of partial gene sequences from bat flies.

Additional file 2. Table S2. GenBank accession numbers, associated species, and country of origin for reference sequences utilized in this study

Additional file 3. Table S3. Nycteribiidae and Streblidae infestation rates categorized by study site, host sex, and physiological status.

Additional file 4. Table S4. Comparison of generalized linear models for bat fly infestation analysis (A) and parameter estimates from the Poisson regression model for bat fly infestation (B)

Additional file 5. Table S5. Results of BLASTN analysis

Additional file 6. Table S6. GenBank accession numbers for sequences deposited from this study.

## Data Availability

The nucleotide sequences obtained in this study were deposited in the GenBank™ database (https://www.ncbi.nlm.nih.gov/nuccore) with the following accession numbers: PQ877438–PQ878457 (cox1) and PQ877927–PQ900123 (28S rRNA). A detailed summary of these sequences is provided in Supplementary Table S6.

## Abbreviations

ABGD: Automatic barcode gap discovery
ASAP: Assemble species by automatic partitioning
BLASTN: Basic local alignment search tool for nucleotide
*Cox1*: Cytochrome oxidase c subunit I
PCR: Polymerase chain reaction
