# Supplementary material for: Host associations and genetic diversity of bat flies (Diptera: Nycteribiidae and Streblidae) in bats from Thailand
Source: Parasit Vectors. 2025 May 24;18:188. doi: 10.1186/s13071-025-06814-y (PMC12103041; doi:10.1186/s13071-025-06814-y)
Supplement: Supplementary file 1 — Additional file 1. Table S1. Oligonucleotide primers and PCR conditions used for the amplification of partial gene sequences from bat flies. [file 13071_2025_6814_MOESM1_ESM.docx]

**Additional file 1: Supplementary Table S1** Oligonucleotide primers and PCR conditions used for the amplification of partial gene sequences from bat flies.

| Gene | Primer | Primer sequence (5' to 3') | PCR condition | Reference |
| --- | --- | --- | --- | --- |
| *Cox1* | LepF1 | ATTCAACCAATCATAAAGATAT | 94 ^ᴼ^C 2 min 1x | Hebert et al. 2004 |
|  | LepR1 | TAAACTTCTGGATGTCCAAAAA | 98 ^ᴼ^C 30 sec  46.8 ^ᴼ^C 30 sec 35x  68 ^ᴼ^C 30 sec |  |
|  |  |  | 68 ^ᴼ^C 10 min 1x |  |
| 28S rRNA | 28S rRNA F2 | AGAGAGAGTTCAAGAGTACGTG | 94 ^ᴼ^C 2 min 1x | Belshaw et al. 2011 |
|  | 28S rRNA 3DR | TAGTTCACCATCTTTCGGGTC | 98 ^ᴼ^C 30 sec  54.5 ^ᴼ^C 30 sec 35x  68 ^ᴼ^C 30 sec |  |
|  |  |  | 68 ^ᴼ^C 10 min 1x |  |

Note: F stands for forward primer and R is reverse primer.

Hebert PDN, Penton EH, Burns JM, Janzen DH, Hallwachs W. Ten species in one: DNA barcoding reveals cryptic species in the neotropical skipper butterfly *Astraptes fulgerator*. Proc Natl Acad Sci U S A. 2004;101:14812–7. <https://doi:10.1073/pnas.0406166101>.

Belshaw R, Lopez-Vaamonde C, Degerli N, Quicke DLJ. Paraphyletic taxa and taxonomic chaining: evaluating the classification of braconine wasps (Hymenoptera: Braconidae) using 28S D2-3 rDNA sequences and morphological characters. Biol J Linn Soc. 2001;73 :411–24. <https://doi:10.1111/j.1095-8312.2001.tb01370.x>.
