# Supplementary material for: Host associations and genetic diversity of bat flies (Diptera: Nycteribiidae and Streblidae) in bats from Thailand
Source: Parasit Vectors. 2025 May 24;18:188. doi: 10.1186/s13071-025-06814-y (PMC12103041; doi:10.1186/s13071-025-06814-y)
Supplement: Supplementary file 2 — Additional file 2. Table S2. GenBank accession numbers, associated species, and country of origin for reference sequences utilized in this study [file 13071_2025_6814_MOESM2_ESM.docx]

**Additional file 2: Supplementary Table S2**. GenBank accession numbers, associated species, and country of origin for reference sequences utilized in this study.

| Accesion number | Species | Country |
| --- | --- | --- |
| OQ184637 | *Raymondia* sp. A | Hong Kong |
| OQ184639 | *Raymondia* sp. B | Hong Kong |
| OQ184638 | *Raymondia* sp. C | Hong Kong |
| OQ184628 | *Brachytarsina amboinensis* isolate GBW13a | Hong Kong |
| OQ184625 | *Brachytarsina amboinensis* isolate CM41 | Hong Kong |
| OQ184626 | *Brachytarsina amboinensis* isolate GBW54a | Hong Kong |
| MT362950 | *Brachytarsina* sp. isolate BE 69 | Korea |
| OM327589 | *Brachytarsina kanoi* voucher BK-3 | Pakistan |
| OM327588 | *Brachytarsina kanoi* voucher BK-2 | Pakistan |
| AB632571 | *Brachytarsina kanoi* | Japan |
| OQ184608 | *Brachytarsina* sp. B GC-2023a isolate IHB120b | Hong Kong |
| OQ184594 | *Brachytarsina* sp. D GC-2023a isolate CHB8 | Hong Kong |
| OQ184589 | *Brachytarsina* sp. C GC-2023a isolate LRb | Hong Kong |
| AB632552 | *Nycteribia pygmaea* isolate NyPy5 | Japan |
| AB632551 | *Nycteribia pygmaea* isolate NyPy4 | Japan |
| AB632550 | *Nycteribia pygmaea* isolate NyPy2 | Japan |
| PP261942 | *Nycteribia kolenatii* voucher L_PS0102 | Belarus |
| PP261941 | *Nycteribia kolenatii* voucher L_PS0101 | Belarus |
| PP261940 | *Nycteribia kolenatii* voucher L_PS0099 | Belarus |
| LC715185 | *Nycteribia* sp. AT36-9 | Japan |
| LC715184 | *Nycteribia* *allotopa* AT36-11 | Japan |
| LC715183 | *Nycteribia allotopa* AT02 | Japan |
| MZ380313 | *Nycteribia schmidlii* isolate H21 | Serbia |
| MZ380312 | *Nycteribia schmidlii* isolate H20 | Serbia |
| MZ380311 | *Nycteribia schmidlii* isolate H19 | Serbia |
| MZ380310 | *Nycteribia schmidlii* isolate H18 | Serbia |
| OQ184588 | *Nycteribia* sp. B GC-2023a isolate GBW28d | Hong Kong |
| OQ184587 | *Nycteribia* sp. B GC-2023a isolate LBW42c | Hong Kong |
| OQ184582 | *Nycteribia* sp. F GC-2023a isolate LBW26c | Hong Kong |
| OQ184581 | *Nycteribia* sp. F GC-2023a isolate LBW55b | Hong Kong |
| OQ184579 | *Nycteribia* sp. D GC-2023a isolate GBW54c | Hong Kong |
| OQ184578 | *Nycteribia* sp. D GC-2023a isolate GBW28e | Hong Kong |
| OQ184577 | *Nycteribia* sp. A GC-2023a isolate RBF30a | Hong Kong |
| OQ184576 | *Nycteribia* sp. A GC-2023a isolate RBF30b | Hong Kong |
| OQ184572 | *Nycteribia* sp. E GC-2023a isolate LBW50d | Hong Kong |
| OQ184570 | *Nycteribia* sp. E GC-2023a isolate LBW75b | Hong Kong |
