# Supplementary material for: Host associations and genetic diversity of bat flies (Diptera: Nycteribiidae and Streblidae) in bats from Thailand
Source: Parasit Vectors. 2025 May 24;18:188. doi: 10.1186/s13071-025-06814-y (PMC12103041; doi:10.1186/s13071-025-06814-y)
Supplement: Supplementary file 3 — Additional file 3. Table S3. Nycteribiidae and Streblidae infestation rates categorized by study site, host sex, and physiological status. [file 13071_2025_6814_MOESM3_ESM.docx]

**Additional file 3: Supplementary Table S3** Nycteribiidae and Streblidae infestation rates categorized by study site, host sex, and physiological status.

| Sample ID | Bat fly family | Number of bat flies | Bat host | Sex | Status | Study site |
| --- | --- | --- | --- | --- | --- | --- |
| THBat19_201 | Nycteribiidae | 1 | *Myotis siligorensis* | M | Adult | MNP |
| THBat19_215 | Nycteribiidae | 1 | *Myotis siligorensis* | M | Adult | MNP |
| THBat19_216 | Nycteribiidae | 1 | *Myotis siligorensis* | M | Adult | MNP |
| THBat20_214 | Nycteribiidae | 1 | *Rhinolophus coelophyllus* | F | Adult | MG |
| THBat20_216 | Nycteribiidae | 2 | *Rhinolophus coelophyllus* | M | Adult | MG |
| THbat22_061 | Nycteribiidae | 1 | *Rhinolophus coelophyllus* | M | Adult | HC |
| THBat20_217 | Nycteribiidae | 1 | *Rhinolophus coelophyllus* | M | Adult | MG |
| THBat20_226 | Nycteribiidae | 2 | *Myotis siligorensis* | M | Adult | MG |
| THBat20_228 | Nycteribiidae | 1 | *Myotis siligorensis* | F | Adult | MG |
| THBat20_231 | Nycteribiidae | 1 | *Myotis siligorensis* | F | Adult | MG |
| THBat20_232 | Nycteribiidae | 2 | *Myotis siligorensis* | F | Adult | MG |
| THBat20_235 | Nycteribiidae | 2 | *Myotis siligorensis* | M | Adult | MG |
| THBat20_238 | Nycteribiidae | 2 | *Myotis siligorensis* | F | Adult | MG |
| THBat20_239 | Nycteribiidae | 4 | *Myotis siligorensis* | M | Adult | MG |
| THBat20_242 | Nycteribiidae | 1 | *Myotis siligorensis* | M | Adult | MG |
| THBat21_114 | Nycteribiidae | 1 | *Myotis siligorensis* | M | Adult | MG |
| THBat21_116 | Nycteribiidae | 1 | *Myotis siligorensis* | M | Adult | MG |
| THBat21_118 | Nycteribiidae | 1 | *Myotis siligorensis* | M | Adult | MG |
| THbat22_056 | Nycteribiidae | 2 | *Myotis siligorensis* | F | Pregnant | HC |
| THbat22_057 | Nycteribiidae | 1 | *Myotis siligorensis* | F | Adult | HC |
| THbat22_058 | Nycteribiidae | 1 | *Myotis siligorensis* | F | Pregnant | HC |
| THbat22_059 | Nycteribiidae | 1 | *Myotis siligorensis* | M | Adult | HC |
| THbat22_060 | Nycteribiidae | 2 | *Myotis siligorensis* | F | Adult | HC |
| THbat22_090 | Nycteribiidae | 1 | *Myotis siligorensis* | M | Adult | KE |
| THbat22_091 | Nycteribiidae | 2 | *Myotis siligorensis* | M | Adult | KE |
| THBat19_167 | Streblidae | 1 | *Taphozous melanopogon* | M | Adult | PC |
| THBat19_168 | Streblidae | 2 | *Taphozous melanopogon* | M | Juvenile | PC |
| THBat19_169 | Streblidae | 2 | *Taphozous melanopogon* | F | Juvenile | PC |
| THBat19170 | Streblidae | 4 | *Taphozous melanopogon* | M | Adult | PC |
| THBat19_172 | Streblidae | 1 | *Hipposideros gentilis* | M | Adult | PC |
| THBat19_173 | Streblidae | 4 | *Hipposideros gentilis* | M | Adult | PC |
| THBat19_178 | Streblidae | 2 | *Taphozous melanopogon* | F | Juvenile | PC |
| THBat19_179 | Streblidae | 4 | *Hipposideros bicolor* | M | Adult | PC |
| THBat19_181 | Streblidae | 2 | *Hipposideros bicolor* | M | Adult | PC |
| THBat19_182 | Streblidae | 3 | *Hipposideros bicolor* | F | Adult | PC |
| THBat19_183 | Streblidae | 5 | *Hipposideros bicolor* | M | Adult | PC |
| THBat19_184 | Streblidae | 1 | *Hipposideros bicolor* | F | Adult | PC |
| THBat19_192 | Streblidae | 1 | *Hipposideros bicolor* | M | Adult | MNP |
| THBat19_193 | Streblidae | 2 | *Hipposideros bicolor* | M | Adult | MNP |
| THBat19_194 | Streblidae | 2 | *Hipposideros bicolor* | F | Parous | MNP |
| THBat19_195 | Streblidae | 1 | *Hipposideros bicolor* | F | Parous | MNP |
| THBat19_197 | Streblidae | 2 | *Hipposideros bicolor* | M | Adult | MNP |
| THBat19_201 | Streblidae | 3 | *Myotis siligorensis* | M | Adult | MNP |
| THBat19_202 | Streblidae | 3 | *Myotis siligorensis* | M | Adult | MNP |
| THBat19_203 | Streblidae | 4 | *Rhinolophus coelophyllus* | M | Adult | MNP |
| THBat19_211 | Streblidae | 1 | *Myotis siligorensis* | F | Adult | MNP |
| THBat19_212 | Streblidae | 3 | *Myotis siligorensis* | M | Adult | MNP |
| THBat19_213 | Streblidae | 2 | *Myotis siligorensis* | M | Adult | MNP |
| THBat19_215 | Streblidae | 1 | *Myotis siligorensis* | M | Adult | MNP |
| THBat19_216 | Streblidae | 1 | *Myotis siligorensis* | M | Adult | MNP |
| THBat19_217 | Streblidae | 1 | *Rhinolophus coelophyllus* | M | Adult | MNP |
| THBat20_014 | Streblidae | 2 | *Taphozous melanopogon* | M | Adult | PC |
| THBat20_015 | Streblidae | 5 | *Taphozous melanopogon* | M | Adult | PC |
| THBat20_016 | Streblidae | 2 | *Taphozous melanopogon* | M | Adult | PC |
| THBat20_018 | Streblidae | 7 | *Taphozous melanopogon* | F | Adult | PC |
| THBat20_020 | Streblidae | 2 | *Taphozous melanopogon* | M | Adult | PC |
| THBat20_021 | Streblidae | 4 | *Taphozous melanopogon* | F | Adult | PC |
| THBat20_023 | Streblidae | 6 | *Taphozous melanopogon* | F | Adult | PC |
| THBat20_024 | Streblidae | 3 | *Taphozous melanopogon* | F | Adult | PC |
| THBat20_025 | Streblidae | 2 | *Taphozous melanopogon* | F | Juvenile | PC |
| THBat20_026 | Streblidae | 4 | *Hipposideros cineraceus* | M | Adult | PC |
| THBat20_037 | Streblidae | 2 | *Taphozous melanopogon* | F | Adult | PC |
| THBat20_039 | Streblidae | 2 | *Hipposideros cineraceus* | F | Adult | PC |
| THBat20_044 | Streblidae | 3 | *Hipposideros cineraceus* | M | Adult | PC |
| THBat20_045 | Streblidae | 2 | *Taphozous melanopogon* | M | Adult | PC |
| THBat20_046 | Streblidae | 8 | *Hipposideros bicolor* | M | Adult | PC |
| THBat20_047 | Streblidae | 28 | *Hipposideros cineraceus* | F | Adult | PC |
| THBat20_048 | Streblidae | 4 | *Hipposideros bicolor* | F | Adult | PC |
| THBat20_049 | Streblidae | 2 | *Taphozous melanopogon* | F | Adult | PC |
| THBat20_050 | Streblidae | 4 | *Taphozous melanopogon* | F | Adult | PC |
| THBat20_052 | Streblidae | 4 | *Taphozous melanopogon* | F | Adult | PC |
| THBat20_053 | Streblidae | 1 | *Taphozous melanopogon* | M | Adult | PC |
| THBat20_054 | Streblidae | 2 | *Hipposideros cineraceus* | M | Adult | PC |
| THBat20_060 | Streblidae | 1 | *Hipposideros cineraceus* | M | Adult | PC |
| THBat20_061 | Streblidae | 2 | *Hipposideros cineraceus* | F | Adult | PC |
| THBat20_063 | Streblidae | 3 | *Hipposideros cineraceus* | M | Adult | PC |
| THBat20_067 | Streblidae | 1 | *Hipposideros cineraceus* | M | Adult | PC |
| THBat20_070 | Streblidae | 4 | *Hipposideros cineraceus* | M | Adult | PC |
| THBat20_071 | Streblidae | 3 | *Hipposideros cineraceus* | M | Adult | PC |
| THBat20_072 | Streblidae | 5 | *Hipposideros cineraceus* | F | Adult | PC |
| THBat20_073 | Streblidae | 3 | *Hipposideros cineraceus* | F | Adult | PC |
| THBat20_083 | Streblidae | 1 | *Hipposideros gentilis* | F | Pregnant | PC |
| THBat20_098 | Streblidae | 2 | *Hipposideros gentilis* | F | Lactating | PC |
| THBat20_099 | Streblidae | 1 | *Hipposideros gentilis* | F | Nonreproductive | PC |
| THBat20_100 | Streblidae | 1 | *Hipposideros gentilis* | F | Pregnant | PC |
| THBat20_103 | Streblidae | 1 | *Taphozous melanopogon* | F | Lactating | PC |
| THBat20_118 | Streblidae | 1 | *Hipposideros gentilis* | F | Lactating | PC |
| THBat20_161 | Streblidae | 3 | *Hipposideros gentilis* | M | Adult | PC |
| THBat20_162 | Streblidae | 2 | *Hipposideros gentilis* | M | Adult | PC |
| THBat20_164 | Streblidae | 1 | *Hipposideros gentilis* | M | Adult | PC |
| THBat20_165 | Streblidae | 3 | *Hipposideros gentilis* | F | Adult | PC |
| THBat20_166 | Streblidae | 3 | *Hipposideros gentilis* | F | Adult | PC |
| THBat20_167 | Streblidae | 1 | *Hipposideros gentilis* | M | Adult | PC |
| THBat20_168 | Streblidae | 1 | *Hipposideros gentilis* | F | Adult | PC |
| THBat20_169 | Streblidae | 3 | *Hipposideros gentilis* | F | Adult | PC |
| THBat20_170 | Streblidae | 4 | *Hipposideros gentilis* | F | Adult | PC |
| THBat20_172 | Streblidae | 2 | *Hipposideros gentilis* | F | Adult | PC |
| THBat20_179 | Streblidae | 1 | *Hipposideros gentilis* | F | Adult | PC |
| THBat20_180 | Streblidae | 4 | *Hipposideros gentilis* | M | Adult | PC |
| THBat20_181 | Streblidae | 3 | *Hipposideros gentilis* | M | Adult | PC |
| THBat20_182 | Streblidae | 1 | *Hipposideros gentilis* | F | Adult | PC |
| THBat20_187 | Streblidae | 1 | *Hipposideros gentilis* | F | Adult | PC |
| THBat20_188 | Streblidae | 1 | *Hipposideros gentilis* | M | Adult | PC |
| THBat20_189 | Streblidae | 1 | *Hipposideros gentilis* | M | Adult | PC |
| THBat20_190 | Streblidae | 2 | *Hipposideros gentilis* | M | Adult | PC |
| THBat20_191 | Streblidae | 1 | *Hipposideros gentilis* | M | Adult | PC |
| THBat20_194 | Streblidae | 8 | *Hipposideros gentilis* | M | Adult | PC |
| THBat20_195 | Streblidae | 3 | *Hipposideros gentilis* | M | Adult | PC |
| THBat20_197 | Streblidae | 12 | *Hipposideros gentilis* | M | Adult | PC |
| THBat20_201 | Streblidae | 1 | *Rhinolophus coelophyllus* | F | Adult | MG |
| THBat20_202 | Streblidae | 2 | *Rhinolophus coelophyllus* | F | Adult | MG |
| THBat20_203 | Streblidae | 1 | *Rhinolophus coelophyllus* | F | Adult | MG |
| THBat20_204 | Streblidae | 1 | *Rhinolophus coelophyllus* | M | Adult | MG |
| THBat20_205 | Streblidae | 1 | *Rhinolophus coelophyllus* | M | Adult | MG |
| THBat20_206 | Streblidae | 1 | *Rhinolophus coelophyllus* | M | Adult | MG |
| THBat20_207 | Streblidae | 2 | *Rhinolophus coelophyllus* | F | Adult | MG |
| THBat20_208 | Streblidae | 2 | *Rhinolophus coelophyllus* | M | Adult | MG |
| THBat20_210 | Streblidae | 3 | *Rhinolophus coelophyllus* | M | Adult | MG |
| THBat20_211 | Streblidae | 1 | *Rhinolophus coelophyllus* | F | Adult | MG |
| THBat20_214 | Streblidae | 1 | *Rhinolophus coelophyllus* | F | Adult | MG |
| THBat20_215 | Streblidae | 4 | *Rhinolophus coelophyllus* | M | Adult | MG |
| THBat20_216 | Streblidae | 2 | *Rhinolophus coelophyllus* | M | Adult | MG |
| THBat20_218 | Streblidae | 3 | *Rhinolophus coelophyllus* | F | Adult | MG |
| THBat20_220 | Streblidae | 2 | *Rhinolophus coelophyllus* | F | Adult | MG |
| THBat20_221 | Streblidae | 1 | *Rhinolophus coelophyllus* | M | Adult | MG |
| THBat20_225 | Streblidae | 2 | *Myotis siligorensis* | M | Adult | MG |
| THBat20_226 | Streblidae | 2 | *Myotis siligorensis* | M | Adult | MG |
| THBat20_228 | Streblidae | 1 | *Myotis siligorensis* | F | Adult | MG |
| THBat20_231 | Streblidae | 4 | *Myotis siligorensis* | F | Adult | MG |
| THBat20_232 | Streblidae | 2 | *Myotis siligorensis* | F | Adult | MG |
| THBat20_233 | Streblidae | 1 | *Myotis siligorensis* | M | Adult | MG |
| THBat20_234 | Streblidae | 2 | *Myotis siligorensis* | F | Adult | MG |
| THBat20_235 | Streblidae | 2 | *Myotis siligorensis* | M | Adult | MG |
| THBat20_239 | Streblidae | 5 | *Myotis siligorensis* | M | Adult | MG |
| THBat20_243 | Streblidae | 2 | *Megaderma spasma* | M | Adult | PC |
| THBat20_244 | Streblidae | 1 | *Hipposideros gentilis* | M | Adult | PC |
| THBat20_245 | Streblidae | 2 | *Hipposideros gentilis* | M | Adult | PC |
| THBat20_246 | Streblidae | 1 | *Hipposideros gentilis* | M | Adult | PC |
| THBat20_247 | Streblidae | 2 | *Hipposideros gentilis* | M | Adult | PC |
| THBat20_250 | Streblidae | 2 | *Hipposideros gentilis* | F | Adult | PC |
| THBat20_251 | Streblidae | 2 | *Hipposideros gentilis* | M | Adult | PC |
| THBat20_252 | Streblidae | 2 | *Hipposideros gentilis* | F | Adult | PC |
| THBat20_253 | Streblidae | 4 | *Hipposideros gentilis* | M | Adult | PC |
| THBat20_259 | Streblidae | 2 | *Taphozous melanopogon* | M | Adult | PC |
| THBat20_261 | Streblidae | 3 | *Taphozous melanopogon* | F | Juvenile | PC |
| THBat20_263 | Streblidae | 2 | *Taphozous melanopogon* | M | Adult | PC |
| THBat20_265 | Streblidae | 1 | *Taphozous melanopogon* | F | Juvenile | PC |
| THBat20_269 | Streblidae | 2 | *Taphozous melanopogon* | M | Juvenile | PC |
| THBat20_270 | Streblidae | 1 | *Taphozous melanopogon* | M | Juvenile | PC |
| THBat20_271 | Streblidae | 1 | *Taphozous melanopogon* | M | Juvenile | PC |
| THBat20_275 | Streblidae | 8 | *Hipposideros gentilis* | M | Adult | PC |
| THBat20_276 | Streblidae | 4 | *Hipposideros gentilis* | M | Adult | PC |
| THBat20_277 | Streblidae | 2 | *Hipposideros gentilis* | F | Parous | PC |
| THBat20_278 | Streblidae | 3 | *Hipposideros gentilis* | F | Parous | PC |
| THBat20_279 | Streblidae | 6 | *Hipposideros gentilis* | M | Adult | PC |
| THBat20_281 | Streblidae | 4 | *Taphozous melanopogon* | F | Adult | PC |
| THBat20_282 | Streblidae | 1 | *Taphozous melanopogon* | F | Adult | PC |
| THBat20_283 | Streblidae | 1 | *Taphozous melanopogon* | M | Adult | PC |
| THBat20_284 | Streblidae | 1 | *Taphozous melanopogon* | M | Adult | PC |
| THBat20_285 | Streblidae | 1 | *Taphozous melanopogon* | M | Adult | PC |
| THBat20_286 | Streblidae | 1 | *Taphozous melanopogon* | M | Adult | PC |
| THBat20_287 | Streblidae | 1 | *Taphozous melanopogon* | F | Adult | PC |
| THBat20_289 | Streblidae | 1 | *Taphozous melanopogon* | M | Adult | PC |
| THBat20_290 | Streblidae | 1 | *hipposideros gentilis* | F | Adult | PC |
| THBat20_291 | Streblidae | 10 | *Hipposideros gentilis* | F | Adult | PC |
| THBat20_292 | Streblidae | 12 | *Hipposideros gentilis* | F | Adult | PC |
| THBat20_293 | Streblidae | 10 | *Hipposideros gentilis* | F | Adult | PC |
| THBat20_294 | Streblidae | 7 | *Hipposideros gentilis* | M | Adult | PC |
| THBat20_295 | Streblidae | 5 | *Hipposideros gentilis* | M | Adult | PC |
| THBat20_296 | Streblidae | 2 | *Hipposideros gentilis* | M | Adult | PC |
| THBat20_297 | Streblidae | 12 | *Hipposideros gentilis* | M | Adult | PC |
| THBat20_298 | Streblidae | 5 | *Hipposideros gentilis* | M | Adult | PC |
| THBat20_299 | Streblidae | 4 | *Hipposideros gentilis* | M | Adult | PC |
| THBat20_300 | Streblidae | 3 | *Rhinolophus coelophyllus* | M | Adult | PC |
| THBat20_301 | Streblidae | 4 | *Rhinolophus coelophyllus* | M | Adult | PC |
| THBat20_302 | Streblidae | 6 | *Rhinolophus coelophyllus* | M | Adult | PC |
| THBat20_303 | Streblidae | 1 | *Rhinolophus coelophyllus* | M | Adult | PC |
| THBat20_306 | Streblidae | 2 | *Hipposideros gentilis* | M | Adult | PC |
| THBat20_307 | Streblidae | 6 | *Hipposideros gentilis* | M | Adult | PC |
| THBat20_308 | Streblidae | 3 | *Hipposideros gentilis* | M | Adult | PC |
| THBat20_309 | Streblidae | 8 | *Hipposideros gentilis* | M | Adult | PC |
| THBat20_311 | Streblidae | 2 | *Hipposideros gentilis* | M | Adult | PC |
| THBat20_312 | Streblidae | 1 | *Hipposideros gentilis* | M | Adult | PC |
| THBat20_314 | Streblidae | 2 | *Hipposideros gentilis* | M | Adult | LRTTS |
| THBat20_316 | Streblidae | 1 | *Hipposideros gentilis* | F | Adult | LRTTS |
| THBat20_317 | Streblidae | 2 | *Rhinolophus coelophyllus* | M | Adult | LRTTS |
| THBat20_318 | Streblidae | 1 | *Rhinolophus coelophyllus* | F | Adult | LRTTS |
| THBat21_037 | Streblidae | 3 | *Hipposideros gentilis* | M | Adult | PC |
| THBat21_038 | Streblidae | 4 | *Hipposideros gentilis* | M | Adult | PC |
| THBat21_040 | Streblidae | 6 | *Hipposideros gentilis* | M | Adult | PC |
| THBat21_041 | Streblidae | 4 | *Hipposideros gentilis* | M | Adult | PC |
| THBat21_042 | Streblidae | 4 | *Hipposideros gentilis* | M | Adult | PC |
| THBat21_043 | Streblidae | 2 | *Hipposideros gentilis* | M | Adult | PC |
| THBat21_044 | Streblidae | 3 | *Hipposideros gentilis* | M | Adult | PC |
| THBat21_045 | Streblidae | 3 | *Hipposideros gentilis* | M | Adult | PC |
| THBat21_046 | Streblidae | 5 | *Hipposideros gentilis* | M | Adult | PC |
| THBat21_047 | Streblidae | 6 | *Hipposideros gentilis* | M | Adult | PC |
| THBat21_048 | Streblidae | 9 | *Hipposideros gentilis* | M | Adult | PC |
| THBat21_049 | Streblidae | 4 | *Hipposideros gentilis* | M | Adult | PC |
| THBat21_050 | Streblidae | 4 | *Hipposideros gentilis* | F | Adult | PC |
| THBat21_051 | Streblidae | 5 | *Hipposideros gentilis* | M | Adult | PC |
| THBat21_053 | Streblidae | 5 | *Hipposideros gentilis* | M | Adult | PC |
| THBat21_054 | Streblidae | 1 | *Hipposideros gentilis* | M | Adult | PC |
| THBat21_057 | Streblidae | 2 | *Hipposideros gentilis* | F | Adult | PC |
| THBat21_058 | Streblidae | 1 | *Hipposideros gentilis* | M | Adult | PC |
| THBat21_060 | Streblidae | 1 | *Hipposideros gentilis* | M | Adult | PC |
| THBat21_062 | Streblidae | 2 | *Hipposideros gentilis* | M | Adult | PC |
| THBat21_064 | Streblidae | 2 | *Hipposideros gentilis* | M | Adult | PC |
| THBat21_066 | Streblidae | 1 | *Hipposideros gentilis* | M | Adult | PC |
| THBat21_067 | Streblidae | 4 | *Hipposideros gentilis* | F | Adult | PC |
| THBat21_068 | Streblidae | 2 | *Hipposideros gentilis* | F | Adult | PC |
| THBat21_071 | Streblidae | 4 | *Hipposideros gentilis* | F | Adult | PC |
| THBat21_072 | Streblidae | 1 | *Craseonycteris thonglongyai* | F | Adult nulliparous | PC |
| THBat21_074 | Streblidae | 1 | *Craseonycteris thonglongyai* | F | Adult | PC |
| THBat21_076 | Streblidae | 1 | *Rhinolophus coelophyllus* | F | Pregnant | PC |
| THBat21_088 | Streblidae | 1 | *Taphozous melanopogon* | F | Adult nulliparous | PC |
| THBat21_096 | Streblidae | 1 | *Taphozous melanopogon* | M | Adult | PC |
| THBat21_106 | Streblidae | 1 | *Rhinolophus coelophyllus* | M | Adult | MG |
| THBat21_107 | Streblidae | 2 | *Rhinolophus coelophyllus* | M | Adult | MG |
| THBat21_108 | Streblidae | 1 | *Rhinolophus coelophyllus* | M | Adult | MG |
| THBat21_110 | Streblidae | 1 | *Rhinolophus coelophyllus* | M | Adult | MG |
| THBat21_111 | Streblidae | 2 | *Rhinolophus coelophyllus* | M | Adult | MG |
| THBat21_114 | Streblidae | 1 | *Myotis siligorensis* | M | Adult | MG |
| THBat21_116 | Streblidae | 1 | *Myotis siligorensis* | M | Adult | MG |
| THBat21_122 | Streblidae | 1 | *Hipposideros armiger* | F | Nulliparous | DWC |
| THBat21_125 | Streblidae | 1 | *Rhinolophus pearsonii* | F | Parous | DWC |
| THbat22_002 | Streblidae | 1 | *Hipposideros gentilis* | M | Adult | PC |
| THbat22_003 | Streblidae | 3 | *Hipposideros gentilis* | M | Adult | PC |
| THbat22_004 | Streblidae | 3 | *Hipposideros gentilis* | M | Adult | PC |
| THbat22_005 | Streblidae | 4 | *Hipposideros gentilis* | M | Adult | PC |
| THbat22_006 | Streblidae | 1 | *Hipposideros gentilis* | M | Adult | PC |
| THbat22_008 | Streblidae | 1 | *Hipposideros gentilis* | M | Parous | PC |
| THbat22_009 | Streblidae | 2 | *Hipposideros gentilis* | M | Adult | PC |
| THbat22_010 | Streblidae | 1 | *Hipposideros gentilis* | M | Nulliparous | PC |
| THbat22_011 | Streblidae | 1 | *Hipposideros gentilis* | M | Adult | PC |
| THbat22_012 | Streblidae | 1 | *Hipposideros gentilis* | M | Adult | PC |
| THbat22_013 | Streblidae | 4 | *Hipposideros gentilis* | M | Nulliparous | PC |
| THbat22_028 | Streblidae | 1 | *Hipposideros gentilis* | M | Adult | TK |
| THbat22_030 | Streblidae | 1 | *Hipposideros gentilis* | F | Parous | TK |
| THbat22_037 | Streblidae | 1 | *Hipposideros gentilis* | M | Adult | TK |
| THbat22_040 | Streblidae | 1 | *Hipposideros gentilis* | M | Adult | PC |
| THbat22_041 | Streblidae | 3 | *Hipposideros gentilis* | M | Adult | PC |
| THbat22_042 | Streblidae | 2 | *Hipposideros gentilis* | F | Parous | PC |
| THbat22_043 | Streblidae | 3 | *Hipposideros gentilis* | M | Adult | PC |
| THbat22_045 | Streblidae | 3 | *Hipposideros gentilis* | M | Adult | PC |
| THbat22_058 | Streblidae | 1 | *Myotis siligorensis* | M | Pregnant | HC |
| THbat22_060 | Streblidae | 2 | *Myotis siligorensis* | M | Adult | HC |
| THbat22_064 | Streblidae | 1 | *Hipposideros gentilis* | M | Pregnant | PC |
| THbat22_065 | Streblidae | 1 | *Hipposideros gentilis* | F | Adult | PC |
| THbat22_066 | Streblidae | 1 | *Hipposideros gentilis* | F | Adult | PC |
| THbat22_068 | Streblidae | 1 | *Hipposideros gentilis* | F | Adult | PC |
| THbat22_070 | Streblidae | 1 | *Hipposideros gentilis* | F | Pregnant | PC |
| THbat22_071 | Streblidae | 2 | *Hipposideros gentilis* | F | Pregnant | PC |
| THbat22_072 | Streblidae | 2 | *Hipposideros gentilis* | F | Parous | PC |
| THbat22_082 | Streblidae | 1 | *Hipposideros gentilis* | F | Pregnant | PC |
| THbat22_088 | Streblidae | 1 | *Myotis siligorensis* | M | Adult | KE |
| THbat22_089 | Streblidae | 3 | *Myotis siligorensis* | M | Adult | KE |
| THbat22_091 | Streblidae | 2 | *Myotis siligorensis* | M | Adult | KE |
| THbat22_095 | Streblidae | 3 | *Hipposideros larvatus* | M | Lactating | KE |
| THbat22_096 | Streblidae | 1 | *Hipposideros atrox* | M | Adult | KE |
| THbat22_097 | Streblidae | 2 | *Hipposideros atrox* | M | Adult | KE |
| THbat22_099 | Streblidae | 3 | *Hipposideros atrox* | M | Adult | KE |
| THbat22_100 | Streblidae | 1 | *Hipposideros gentilis* | M | Adult | PC |
| THbat22_102 | Streblidae | 1 | *Hipposideros gentilis* | F | Adult | PC |
| THbat22_103 | Streblidae | 1 | *Hipposideros gentilis* | F | Adult | PC |
| THbat22_104 | Streblidae | 1 | *Hipposideros gentilis* | M | Adult | PC |
| THbat22_105 | Streblidae | 1 | *Hipposideros gentilis* | M | Adult | PC |
| THbat22_106 | Streblidae | 2 | *Hipposideros gentilis* | M | Adult | PC |
| THbat22_107 | Streblidae | 1 | *Hipposideros gentilis* | M | Adult | PC |
| THbat22_110 | Streblidae | 1 | *Hipposideros gentilis* | M | Adult | PC |
| THbat22_114 | Streblidae | 2 | *Hipposideros gentilis* | F | Adult | PC |
| THbat22_115 | Streblidae | 1 | *Hipposideros gentilis* | M | Adult | PC |
| THbat22_116 | Streblidae | 3 | *Hipposideros gentilis* | F | Adult | PC |
| THbat22_117 | Streblidae | 3 | *Rhinolophus malayanus* | M | Adult | PC |
| THbat22_120 | Streblidae | 1 | *Hipposideros gentilis* | M | Adult | PC |
| THbat22_126 | Streblidae | 4 | *Hipposideros gentilis* | F | Adult | PC |
| THbat22_128 | Streblidae | 1 | *Hipposideros gentilis* | M | Adult | PC |
| THbat22_129 | Streblidae | 1 | *Hipposideros gentilis* | F | Adult | PC |
| THbat22_130 | Streblidae | 1 | *Hipposideros gentilis* | M | Adult | PC |
| THbat22_131 | Streblidae | 3 | *Hipposideros gentilis* | M | Adult | PC |
| THbat22_138 | Streblidae | 1 | *Hipposideros gentilis* | M | Adult | PC |
| THbat22_151 | Streblidae | 2 | *Hipposideros gentilis* | F | Adult | PC |
| THbat22_152 | Streblidae | 1 | *Hipposideros gentilis* | M | Adult | PC |
| THbat22_154 | Streblidae | 4 | *Hipposideros gentilis* | M | Adult | PC |
| THbat22_155 | Streblidae | 1 | *Hipposideros gentilis* | F | Adult | PC |
| THbat22_157 | Streblidae | 4 | *Hipposideros gentilis* | F | Adult | PC |
| THbat22_177 | Streblidae | 2 | *Taphozous melanopogon* | F | Adult | PC |
| THbat22_179 | Streblidae | 2 | *Taphozous melanopogon* | F | Adult | PC |
| THbat22_181 | Streblidae | 1 | *Taphozous melanopogon* | F | Adult | PC |
| THbat22_185 | Streblidae | 2 | *Hipposideros gentilis* | F | Adult | PC |
| THbat22_186 | Streblidae | 2 | *Hipposideros gentilis* | F | Adult | PC |
| THbat22_187 | Streblidae | 4 | *Hipposideros gentilis* | F | Adult | PC |
| THbat22_188 | Streblidae | 3 | *Hipposideros gentilis* | F | Adult | PC |
| THbat22_196 | Streblidae | 1 | *Rhinolophus coelophyllus* | F | Adult | MG |
| THbat22_197 | Streblidae | 3 | *Rhinolophus coelophyllus* | M | Adult | MG |
| THbat22_201 | Streblidae | 1 | *Rhinolophus coelophyllus* | M | Adult | MG |
| THbat22_207 | Streblidae | 3 | *Rhinolophus coelophyllus* | M | Adult | MG |
| THbat22_208 | Streblidae | 6 | *Rhinolophus coelophyllus* | F | Adult | MG |
| THbat22_209 | Streblidae | 2 | *Rhinolophus coelophyllus* | F | Adult | MG |
| THbat22_211 | Streblidae | 1 | *Hipposideros gentilis* | F | Adult | PC |
| THbat22_212 | Streblidae | 1 | *Megaderma spasma* | M | Adult | PC |
| THbat22_215 | Streblidae | 2 | *Hipposideros gentilis* | M | Adult | PC |
| THbat22_216 | Streblidae | 2 | *Hipposideros gentilis* | F | Adult | PC |
| THbat22_217 | Streblidae | 4 | *Hipposideros gentilis* | M | Adult | PC |
| THbat22_219 | Streblidae | 1 | *Hipposideros gentilis* | F | Adult | PC |
| THbat22_220 | Streblidae | 1 | *Hipposideros gentilis* | M | Adult | PC |
| THbat22_221 | Streblidae | 3 | *Taphozous melanopogon* | F | Adult | PC |
| THbat22_224 | Streblidae | 1 | *Hipposideros gentilis* | M | Adult | PC |
| THbat22_225 | Streblidae | 2 | *Hipposideros gentilis* | F | Adult | PC |
| THbat22_226 | Streblidae | 1 | *Hipposideros gentilis* | M | Adult | PC |
| THbat22_227 | Streblidae | 2 | *Hipposideros gentilis* | M | Adult | PC |
| THbat22_232 | Streblidae | 1 | *Taphozous melanopogon* | M | Adult | PC |
| THbat22_235 | Streblidae | 1 | *Taphozous melanopogon* | F | Adult | PC |
| THbat22_237 | Streblidae | 2 | *Taphozous melanopogon* | M | Adult | PC |
| THbat22_238 | Streblidae | 1 | *Taphozous melanopogon* | F | Adult | PC |

DWC: Daowadung Cave, HC: Hintok Cutting, KE: Khanom Electricity, LRTSS: Lainan Research and Technology Transfer Station, MG: Ma Gleua, MNP: Ma Now Phee, PC: Phra Cave and TC: Ta-Klor Cave
