# Supplementary material for: Host associations and genetic diversity of bat flies (Diptera: Nycteribiidae and Streblidae) in bats from Thailand
Source: Parasit Vectors. 2025 May 24;18:188. doi: 10.1186/s13071-025-06814-y (PMC12103041; doi:10.1186/s13071-025-06814-y)
Supplement: Supplementary file 4 — Additional file 4. Table S4. Comparison of generalized linear models for bat fly infestation analysis (A) and parameter estimates from the Poisson regression model for bat fly infestation (B) [file 13071_2025_6814_MOESM4_ESM.docx]

**Additional file 4: Table S4.** Comparison of Generalized Linear Models for bat fly infestation analysis **(A)** and parameter estimates from the Poisson regression model for bat fly infestation **(B)**.

The Poisson Generalized Linear Model (GLM) formula used in this analysis is:


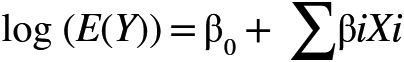


*E(Y*) is the expected number of bat flies (infestation count), β_0_ is the intercept, *Xi* represents the predictor variables (bat species, sex, physiological status, and study site),​*β_i_* are the coefficients estimated for each predictor.

*GLM formula for this study*

For each infestation type, the Poisson regression model is:


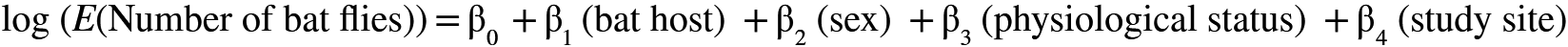


Dependent variable/ response (Y): number of bat flies

Independent variables/ predictor (X): bat host, sex, physiological status, and study site

**Table S4.** Comparison of generalized linear model for bat fly infestation analysis **(A)** and Parameter estimates from the Poisson regression model for bat fly infestation **(B)**.

**(A)**

| Distribution | Streblid infestation | | Nycteribiid infestation | | Total Infestation | |
| --- | --- | --- | --- | --- | --- | --- |
|  | AIC | BIC | AIC | BIC | AIC | BIC |
| Poisson* | 1172.31 | -1131.57 | 76.21 | -51.90 | 1239.51 | -1286.13 |
| Negative Binomial | 1257.05 | -1405.94 | 96.15 | -55.70 | 1341.20 | -1567.28 |

AIC: Akaike Information Criteria, BIC: Bayesian Information Criteria. *Best model based on selection criteria.

**(B)**

| Model | Predictor | Estimate | Std. Error | Wald χ2 | P-value | Significance |
| --- | --- | --- | --- | --- | --- | --- |
| Nycteribiid Infestation | Intercept | 0.06428 | 0.26560 | 0.05857 | 0.80877 | Not significant |
| Nycteribiid Infestation | Q('Bat host')[T.Hipposideros armiger] | 0.00000 | 0.00000 | 0.06438 | 0.79971 | Not significant |
| Nycteribiid Infestation | Q('Bat host')[T.Hipposideros atrox] | 0.00000 | 0.00000 | 0.10544 | 0.74540 | Not significant |
| Nycteribiid Infestation | Q('Bat host')[T.Hipposideros bicolor] | 0.00000 | 0.00000 | 0.12361 | 0.72515 | Not significant |
| Nycteribiid Infestation | Q('Bat host')[T.Hipposideros cineraceus] | 0.00000 | 0.00000 | 0.11903 | 0.73008 | Not significant |
| Nycteribiid Infestation | Q('Bat host')[T.Hipposideros gentilis] | 0.00000 | 0.00000 | 0.04877 | 0.82522 | Not significant |
| Nycteribiid Infestation | Q('Bat host')[T.Hipposideros larvatus] | 0.00000 | 0.00000 | 0.03592 | 0.84968 | Not significant |
| Nycteribiid Infestation | Q('Bat host')[T.Megaderma spasma] | 0.00000 | 0.00000 | 0.00000 | 0.00000 | Not significant |
| Nycteribiid Infestation | Q('Bat host')[T.Myotis siligorensis] | 0.14949 | 0.21353 | 0.49013 | 0.48387 | Not significant |
| Nycteribiid Infestation | Q('Bat host')[T.Rhinolophus coelophyllus] | -0.08521 | 0.33865 | 0.06331 | 0.80134 | Not significant |
| Nycteribiid Infestation | Q('Bat host')[T.Rhinolophus malayanus] | 0.00000 | 0.00000 | 0.00000 | 0.00000 | Not significant |
| Nycteribiid Infestation | Q('Bat host')[T.Rhinolophus pearsonii] | 0.00000 | 0.00000 | 0.00000 | 0.00000 | Not significant |
| Nycteribiid Infestation | Q('Bat host')[T.Taphozous melanopogon] | 0.00000 | 0.00000 | 0.00000 | 0.00000 | Not significant |
| Nycteribiid Infestation | Sex[T.M] | 0.08322 | 0.40558 | 0.04210 | 0.83742 | Not significant |
| Nycteribiid Infestation | Status[T.Adult nulliparous] | 0.00000 | 0.00000 | 0.00000 | 0.00000 | Not significant |
| Nycteribiid Infestation | Status[T.Juvenile] | 0.00000 | 0.00000 | 0.00000 | 0.00000 | Not significant |
| Nycteribiid Infestation | Status[T.Lactating] | 0.00000 | 0.00000 | 0.00000 | 0.00000 | Not significant |
| Nycteribiid Infestation | Status[T.Nonreproductive] | 0.00000 | 0.00000 | 0.00000 | 0.00000 | Not significant |
| Nycteribiid Infestation | Status[T.Nulliparous] | 0.00000 | 0.00000 | 0.00000 | 0.00000 | Not significant |
| Nycteribiid Infestation | Status[T.Parous] | 0.00000 | 0.00000 | 0.00000 | 0.00000 | Not significant |
| Nycteribiid Infestation | Status[T.Pregnant] | 0.16879 | 0.76130 | 0.04915 | 0.82454 | Not significant |
| Nycteribiid Infestation | Q('Study site')[T.HC] | 0.02291 | 0.40397 | 0.00322 | 0.95477 | Not significant |
| Nycteribiid Infestation | Q('Study site')[T.KE] | 0.10848 | 0.50833 | 0.04554 | 0.83102 | Not significant |
| Nycteribiid Infestation | Q('Study site')[T.LRTTS] | 0.00000 | 0.00000 | 0.00000 | 0.00000 | Not significant |
| Nycteribiid Infestation | Q('Study site')[T.MG] | 0.22988 | 0.26082 | 0.77682 | 0.37812 | Not significant |
| Nycteribiid Infestation | Q('Study site')[T.MNP] | -0.29699 | 0.50833 | 0.34135 | 0.55905 | Not significant |
| Nycteribiid Infestation | Q('Study site')[T.PC] | 0.00000 | 0.00000 | 0.00000 | 0.00000 | Not significant |
| Nycteribiid Infestation | Q('Study site')[T.TK] | 0.00000 | 0.00000 | 0.00000 | 0.00000 | Not significant |
| Streblid Infestation | Intercept | -0.18124 | 0.57051 | 0.10092 | 0.75072 | Not significant |
| Streblid Infestation | Q('Bat host')[T.Hipposideros armiger] | 0.29555 | 1.13747 | 0.06751 | 0.79499 | Not significant |
| Streblid Infestation | Q('Bat host')[T.Hipposideros atrox] | 1.18231 | 0.99342 | 1.41644 | 0.23399 | Not significant |
| Streblid Infestation | Q('Bat host')[T.Hipposideros bicolor] | 1.12935 | 0.77818 | 2.10622 | 0.14670 | Not significant |
| Streblid Infestation | Q('Bat host')[T.Hipposideros cineraceus] | 1.35690 | 0.76718 | 3.12819 | 0.07695 | Not significant |
| Streblid Infestation | Q('Bat host')[T.Hipposideros gentilis] | 0.89054 | 0.75848 | 1.37852 | 0.24035 | Not significant |
| Streblid Infestation | Q('Bat host')[T.Hipposideros larvatus] | 2.37108 | 1.18820 | 3.98212 | 0.04599 | **Significant (p=0.046)** |
| Streblid Infestation | Q('Bat host')[T.Megaderma spasma] | 0.26542 | 0.95330 | 0.07752 | 0.78069 | Not significant |
| Streblid Infestation | Q('Bat host')[T.Myotis siligorensis] | 1.18231 | 0.80850 | 2.13846 | 0.14365 | Not significant |
| Streblid Infestation | Q('Bat host')[T.Rhinolophus coelophyllus] | 1.10838 | 0.78919 | 1.97247 | 0.16019 | Not significant |
| Streblid Infestation | Q('Bat host')[T.Rhinolophus malayanus] | 0.95857 | 0.95344 | 1.01079 | 0.31471 | Not significant |
| Streblid Infestation | Q('Bat host')[T.Rhinolophus pearsonii] | 0.59037 | 1.08426 | 0.29647 | 0.58611 | Not significant |
| Streblid Infestation | Q('Bat host')[T.Taphozous melanopogon] | 0.62009 | 0.76000 | 0.66570 | 0.41455 | Not significant |
| Streblid Infestation | Sex[T.M] | -0.12268 | 0.08046 | 2.32520 | 0.12729 | Not significant |
| Streblid Infestation | Status[T.Adult nulliparous] | -0.62009 | 0.75973 | 0.66617 | 0.41439 | Not significant |
| Streblid Infestation | Status[T.Juvenile] | -0.25477 | 0.27362 | 0.86700 | 0.35179 | Not significant |
| Streblid Infestation | Status[T.Lactating] | -0.78330 | 0.50457 | 2.41004 | 0.12056 | Not significant |
| Streblid Infestation | Status[T.Nonreproductive] | -1.15327 | 1.00346 | 1.32089 | 0.25043 | Not significant |
| Streblid Infestation | Status[T.Nulliparous] | -0.11429 | 0.45103 | 0.06422 | 0.79995 | Not significant |
| Streblid Infestation | Status[T.Parous] | -0.40913 | 0.28174 | 2.10864 | 0.14647 | Not significant |
| Streblid Infestation | Status[T.Pregnant] | -1.01305 | 0.34415 | 8.66484 | 0.00324 | **Significant (p=0.003)** |
| Streblid Infestation | Q('Study site')[T.HC] | -0.08954 | 0.62385 | 0.02060 | 0.88588 | Not significant |
| Streblid Infestation | Q('Study site')[T.KE] | -0.18523 | 0.50083 | 0.13679 | 0.71149 | Not significant |
| Streblid Infestation | Q('Study site')[T.LRTTS] | -0.35921 | 0.47520 | 0.57139 | 0.44971 | Not significant |
| Streblid Infestation | Q('Study site')[T.MG] | -0.21010 | 0.33454 | 0.39440 | 0.52999 | Not significant |
| Streblid Infestation | Q('Study site')[T.MNP] | -0.16712 | 0.35157 | 0.22596 | 0.63454 | Not significant |
| Streblid Infestation | Q('Study site')[T.PC] | 0.44397 | 0.31150 | 2.03134 | 0.15408 | Not significant |
| Streblid Infestation | Q('Study site')[T.TK] | -0.49994 | 0.60366 | 0.68588 | 0.40757 | Not significant |
| Total Infestation | Intercept | -0.20505 | 0.56774 | 0.13045 | 0.71797 | Not significant |
| Total Infestation | Q('Bat host')[T.Hipposideros armiger] | 0.31857 | 1.13786 | 0.07839 | 0.77950 | Not significant |
| Total Infestation | Q('Bat host')[T.Hipposideros atrox] | 1.18365 | 0.95939 | 1.52213 | 0.21730 | Not significant |
| Total Infestation | Q('Bat host')[T.Hipposideros bicolor] | 1.13417 | 0.77843 | 2.12284 | 0.14512 | Not significant |
| Total Infestation | Q('Bat host')[T.Hipposideros cineraceus] | 1.35304 | 0.76764 | 3.10676 | 0.07797 | Not significant |
| Total Infestation | Q('Bat host')[T.Hipposideros gentilis] | 0.88245 | 0.75902 | 1.35168 | 0.24498 | Not significant |
| Total Infestation | Q('Bat host')[T.Hipposideros larvatus] | 2.36469 | 1.15873 | 4.16471 | 0.04127 | **Significant (p=0.041)** |
| Total Infestation | Q('Bat host')[T.Megaderma spasma] | 0.25807 | 0.95398 | 0.07318 | 0.78676 | Not significant |
| Total Infestation | Q('Bat host')[T.Myotis siligorensis] | 1.07829 | 0.80241 | 1.80581 | 0.17901 | Not significant |
| Total Infestation | Q('Bat host')[T.Rhinolophus coelophyllus] | 1.07235 | 0.78953 | 1.84475 | 0.17439 | Not significant |
| Total Infestation | Q('Bat host')[T.Rhinolophus malayanus] | 0.95126 | 0.95540 | 0.99136 | 0.31941 | Not significant |
| Total Infestation | Q('Bat host')[T.Rhinolophus pearsonii] | 0.60072 | 1.08765 | 0.30505 | 0.58073 | Not significant |
| Total Infestation | Q('Bat host')[T.Taphozous melanopogon] | 0.61705 | 0.76057 | 0.65821 | 0.41719 | Not significant |
| Total Infestation | Sex[T.M] | -0.11426 | 0.07883 | 2.10101 | 0.14720 | Not significant |
| Total Infestation | Status[T.Adult nulliparous] | -0.61702 | 0.76269 | 0.65449 | 0.41851 | Not significant |
| Total Infestation | Status[T.Juvenile] | -0.25416 | 0.27431 | 0.85848 | 0.35417 | Not significant |
| Total Infestation | Status[T.Lactating] | -0.77554 | 0.50422 | 2.36570 | 0.12403 | Not significant |
| Total Infestation | Status[T.Nonreproductive] | -1.14410 | 1.00503 | 1.29589 | 0.25497 | Not significant |
| Total Infestation | Status[T.Nulliparous] | -0.11348 | 0.45360 | 0.06259 | 0.80245 | Not significant |
| Total Infestation | Status[T.Parous] | -0.39561 | 0.28207 | 1.96706 | 0.16076 | Not significant |
| Total Infestation | Status[T.Pregnant] | -0.83520 | 0.30209 | 7.64389 | 0.00570 | **Significant (p=0.006)** |
| Total Infestation | Q('Study site')[T.HC] | -0.25451 | 0.42101 | 0.36545 | 0.54550 | Not significant |
| Total Infestation | Q('Study site')[T.KE] | -0.17117 | 0.44445 | 0.14833 | 0.70014 | Not significant |
| Total Infestation | Q('Study site')[T.LRTTS] | -0.31587 | 0.46982 | 0.45201 | 0.50138 | Not significant |
| Total Infestation | Q('Study site')[T.MG] | -0.18793 | 0.32521 | 0.33394 | 0.56335 | Not significant |
| Total Infestation | Q('Study site')[T.MNP] | -0.18376 | 0.34403 | 0.28531 | 0.59324 | Not significant |
| Total Infestation | Q('Study site')[T.PC] | 0.46671 | 0.30598 | 2.32655 | 0.12718 | Not significant |
| Total Infestation | Q('Study site')[T.TK] | -0.47782 | 0.60241 | 0.62913 | 0.42767 | Not significant |

Q= respon/number of bat fly, T=variable category as predictor
